# From Reinforcement to Prediction: Mechanical Behavior and Modeling of CNT-Reinforced Cementitious Composites

**DOI:** 10.3390/nano16211371

**Published:** 2026-10-27

**Authors:** Nicolò Rimondotto, Alice Rossi, Matteo Pavese, Luca Lavagna

**Affiliations:** Department of Applied Science and Technology (DISAT), Politecnico di Torino, c.so Duca degli Abruzzi 24, 10129 Torino, Italy; nicolo.rimondotto@polito.it (N.R.); alice_rossi@polito.it (A.R.); matteo.pavese@polito.it (M.P.)

**Keywords:** cement, composites, CNTs, mechanical reinforcement, modeling

## Abstract

Cement-based materials are among the most widely used substances in the construction industry owing to their widespread availability and reliable performance. However, their quasi-brittle behavior and limited tensile capacity often lead to the initiation and propagation of microcracks, which can compromise durability and structural integrity. In this context, the incorporation of carbon nanotubes (CNTs), owing to their exceptional stiffness and strength, has emerged as a promising strategy to enhance the mechanical performance of cementitious matrices and mitigate their intrinsic brittleness. This comprehensive review critically assesses the reinforcing mechanisms associated with CNT addition, with particular emphasis on hydration promotion and nucleation effects, pore-filling and microstructural densification, CNT–matrix interfacial interaction and crack propagation inhibition. Furthermore, this work critically examines modeling approaches for predicting the mechanical properties of CNT-reinforced cementitious composites, including analytical formulations, numerical methods and recent advances based on machine learning and artificial intelligence. This review aims to consolidate the current state of knowledge while highlighting current limitations and outlining future research directions for the development of high-performance cementitious composites.

## 1. Introduction

Cementitious materials are a class of inorganic binders that undergo hydration reactions upon exposure to water, forming a paste that progressively hardens into a solid matrix [1]. Among these, ordinary Portland cement (OPC) is the most widely used and industrially significant, serving as the primary binding phase in concrete [2,3]. OPC is mainly composed of tricalcium silicate (3CaO∙SiO_2_, C_3_S in cement chemistry notation), along with dicalcium silicate (2CaO∙SiO_2_, C_2_S), tricalcium aluminate (3CaO∙Al_2_O_3_, C_3_A) and tetracalcium aluminoferrite (4CaO∙Al_2_O_3_∙Fe_2_O_3_, C_4_AF) [4]. Upon hydration, C_3_S and C_2_S predominantly react with water to form calcium silicate hydrate (C-S-H) gel and calcium hydroxide, which are primarily responsible for strength development, whereas C_3_A and C_4_AF contribute to the formation of various secondary hydration products [5].

Cementitious materials encompass different material systems, including cement paste, mortar, conventional concrete and ultra-high-performance concrete (UHPC), which differ in composition, microstructure and mechanical behavior. Cement paste primarily consists of cement, water and any incorporated admixtures, whereas mortar additionally contains fine aggregates, and conventional concrete contains both fine and coarse aggregates [6,7]. In contrast, UHPC is characterized by an optimal mixture design, typically involving a low water/cement ratio and an optimized gradation of granular constituents, to achieve high mechanical strength and superior durability [8,9].

Cementitious materials are extensively employed in construction due to their favorable compressive strength, stiffness, and durability, along with the widespread availability of raw materials. However, they inherently exhibit low tensile strength and quasi-brittle behavior, which limits their performance in applications requiring resistance to cracking and fracture [10]. To address these limitations, the incorporation of nanofillers has emerged as a promising strategy. Nanofillers can enhance the mechanical properties of cement-based systems through multiple mechanisms, including acceleration of hydration reactions, refinement of pore structure, densification of the microstructure and the promotion of additional C-S-H gel formation, among others [11].

Among nanoscale additives, carbon nanotubes (CNTs) have attracted significant attention owing to their exceptional mechanical, electrical and thermal properties [12]. First reported by Iijima [13] in 1991, CNTs are synthetized in two primary morphologies: single-walled CNTs (SWCNTs) and multi-walled CNTs (MWCNTs). SWCNTs consist of a single graphene sheet rolled into a cylindrical structure, whereas MWCNTs comprise multiple concentric graphene cylinders [14]. CNTs typically exhibit diameters on the order of nanometers and lengths up to micrometers [15]. Owing to their nanoscale dimensions and unique tubular geometries, CNTs can significantly enhance the mechanical performance of cementitious systems at ambient temperature [16]. With a Young’s modulus exceeding 1 TPa and tensile strength in the range of 10–100 GPa, CNTs exhibit extraordinary stiffness and strength, making them highly promising candidates for cementitious composites [17,18].

Despite extensive research, a comprehensive understanding of the interplay between CNT dispersion, interfacial interactions, and predictive modeling remains limited. In particular, the integration of multiscale reinforcing mechanisms with data-driven modeling approaches has not yet been systematically addressed.

This work aims to provide a comprehensive insight into the reinforcing effects of CNTs in cementitious matrices, with particular emphasis on the mechanisms responsible for the enhancement of mechanical properties. The following aspects are critically reviewed: (i) the promotion of hydration reactions and the nucleation effect induced by CNTs; (ii) pore-filling and microstructural densification of the cementitious composites; (iii) interfacial interactions between CNTs and the cement matrix and (iv) mechanisms governing crack propagation inhibition.

Furthermore, a dedicated section addresses existing approaches for predicting the mechanical properties of CNT-reinforced cementitious systems, with particular emphasis on compressive strength, flexural strength, and elastic modulus, which are key indicators of their mechanical performance. Analytical formulations, numerical methods and recent advances based on machine learning and artificial intelligence are examined in terms of their predictive capabilities, underlying assumptions and limitations.

### Literature Search Strategy and Study Selection

A literature search was conducted in Scopus and Web of Science to identify studies on the reinforcing mechanisms and mechanical property prediction of CNT-reinforced cementitious composites. The search covered publications from January 2010 to July 2026. The search strategy combined terms related to cementitious materials, carbon nanotubes, mechanical properties, reinforcement mechanisms, analytical and numerical modeling, and machine learning. The terms included “cement”, “mortar”, “concrete”, “carbon nanotubes”, “CNT”, “compressive strength”, “flexural strength”, “tensile strength”, “elastic modulus”, “crack bridging”, “finite element method”, “prediction”, “machine learning” and “artificial neural network”.

Studies were screened by examining their titles and abstracts, followed by full-text assessment of potentially relevant publications. Additional studies were identified by examining the reference lists of selected articles. Eligible studies were those addressing at least one of the following topics: (i) experimental evidence concerning CNT-reinforcement mechanisms in cementitious matrices; (ii) the influence of CNT characteristics, dosage, dispersion and functionalization, or mixture composition on mechanical performance; (iii) analytical, numerical or data-driven models for predicting the mechanical properties of CNT-reinforced cementitious materials.

Studies were excluded when they did not address cementitious matrices, did not investigate CNT-related reinforcement or mechanical properties prediction, or did not provide sufficient information relevant to the scope of this review. For each included study, information was extracted on the material system, CNT characteristics and dosage, dispersion and functionalization procedures, mixture composition and curing conditions, mechanical properties investigated, modeling approach, validation procedure and reported limitations. The findings were then compared to identify recurring trends, conflicting results and methodological limitations.

## 2. Reinforcing Behavior in CNT/Cement Composites

The incorporation of CNTs into cementitious matrices has gained increasing attention due to their potential to enhance the mechanical properties of cement-based composite materials. Several studies [19,20,21,22,23,24,25,26,27,28,29] have shown how the addition of CNTs positively affects engineering properties such as compressive strength, tensile strength, flexural strength and elastic modulus. However, reinforcing efficiency strongly depends on various factors, such as CNTs characteristics (i.e., type, treatment, aspect ratio), content, dispersion quality and bond strength with the matrix [21,30].

Owing to their hydrophobic properties, high surface area and strong van der Waals forces, CNTs tend to agglomerate in aqueous media, leading to the formation of bundles [31,32,33]. The scarce dispersion negatively impacts the composite’s mechanical properties, since the agglomerates act as defect sites and crack initiators in the cement matrix [34,35]. Moreover, a poor dispersion of CNTs raises the viscosity of the cement paste, which leads to increased air entrapment and subsequent higher porosity in the hardened CNT/cement composite [34]. On the other hand, an adequate dispersion guarantees a higher interfacial contact area, as well as effective stress transfer between the matrix and the CNTs [35].

Therefore, dispersion represents a key step in the preparation process of CNT/cement composites. A uniform dispersion can be achieved following three main steps [36]: (i) wetting of the CNTs by the aqueous medium, (ii) deagglomeration of the CNT bundles and (iii) stabilization of the suspension in order to prevent re-agglomeration. The main dispersion processes include mechanical agitation and chemical treatment approaches. Mechanical methods, which can be achieved via ultrasonication [37,38] or high-shear emulsification [36,39], can produce dispersion only if combined with chemical functionalization techniques [35,36]. Chemical approaches can be categorized as chemical (covalent) or physical (non-covalent) methods [35]. Covalent methods provide covalent bonding of functional groups with the CNT surface by exploiting aggressive chemicals, such as pure acids (H_2_SO_4_ or HNO_3_) [40,41]. The addition of functional groups prevents CNT agglomeration but might, as well, introduce structural defects which hinder their reinforcing efficiency [35]. Conversely, physical methods rely on the use of surfactants, which interact with the CNT surface without disrupting their covalent bonds, thus preserving their intrinsic properties [35]. More specifically, surfactants guarantee CNT dispersion, exploiting hydrophobic/hydrophilic interactions: the surfactants’ hydrophobic tails adsorb on the CNT surface, while their hydrophilic heads interact with water [42,43].

Despite the importance of CNT dispersion, its evaluation remains challenging because different studies employ different characterization techniques and assessment criteria. In aqueous suspensions, UV–visible spectroscopy is used to assess dispersion and stability through absorbance measurements [44,45], while optical microscopy can reveal the state of CNT dispersion and the presence of large agglomerates [45]. In the presence of a solvent or dispersant, thermogravimetric analysis (TGA) may also provide information on CNT concentration [46]. By contrast, in hardened cementitious matrices, scanning electron microscopy (SEM), coupled with energy-dispersive X-ray spectroscopy (EDS) and transmission electron microscopy (TEM), is used to examine the local distribution of CNTs and the presence of agglomerates. Confocal Raman microspectroscopy (CRM) has also been employed to investigate the spatial distribution of CNTs in cementitious composites [47]. However, these techniques differ in spatial resolution, sampling scale and the ability to provide quantitative measurements of dispersion. Consequently, dispersion quality reported across different studies cannot generally be considered directly comparable, particularly when different characterization methods, sample preparation procedures and evaluation criteria are employed.

A further concern is the secondary agglomeration of CNTs in the cementitious system occurring after initial dispersion. To address this challenge, in situ growth techniques, which enable the direct synthesis of CNTs on the surface of cement particles, have been suggested as a promising strategy [48,49,50,51].

In addition to dispersion, the concentration of CNTs represents another key parameter influencing composite performance. The existence of a critical incorporation concentration (CIC), typically reported in the range of 0.5–1.0 wt%, implies that CNT content exceeding this threshold can lead to the formation of agglomerates (Figure 1), which may act as crack initiation sites. Furthermore, CNT content above the CIC may hinder cement hydration and, as reported in literature [20,29], increase entrapped air voids within the matrix, ultimately deteriorating composite properties.

Provided that adequate dispersion and optimal filler content are ensured, CNTs effectively contribute to the enhancement of the CNT/cement composite’s mechanical properties through several mechanisms (Figure 2), including [24,30]:

- Promotion of the development and distribution of hydration products due to the nucleation effect of CNTs.
- Pore-filling and formation of a denser cement-based material.
- Strengthened filler–matrix interfacial interaction and improved stress transfer within the CNT/cement composite.
- Inhibition of crack growth and propagation.

### 2.1. Influence of CNTs on the Nucleation Process During Cement Hydration

The formation of a distributed network of CNTs within the matrix represents a key factor in improving composite performance [52]. Owing to their large specific surface area, CNTs provide abundant sites for the precipitation of cement hydration products. This effect may lead to a well-packed arrangement of hydrates and the formation of a denser C-S-H network [53]. Furthermore, CNTs have been observed to accelerate the cement hydration reaction, most likely due to their nucleation effect [21,52,54,55,56,57]. Although chemically inert and not consumed in the reaction, they can promote the kinetics of hydration by acting as nucleation sites for the formation of C-S-H and Ca(OH)_2_. Depending on whether the CNTs are unfunctionalized or functionalized, different mechanisms can contribute to the nucleation process.

Makar et al. [54] provided evidence of nucleation occurring on unfunctionalized CNTs within an ordinary Portland cement (OPC) matrix. As shown in Figure 3, in the as-delivered OPC, large areas of the grain surface remain free of C-S-H after 135 min of hydration. By contrast, as evidenced in Figure 4, CNTs appear to be progressively coated with cementitious products after 60, 135, 180 and 240 min of hydration, ultimately developing a final interconnected meshwork structure. This observation suggests that nucleation may occur either continuously along the entire length of the tubes or at discrete sites, followed by rapid growth of the hydration product along the CNTs until they are fully coated with C-S-H. Additional work by Makar et al. [56] demonstrated the preferential nucleation on CNTs of C-S-H with respect to Ca(OH)_2_, suggesting that, in the case of unfunctionalized CNTs, the predominant nucleation mechanism is electrostatic in nature, promoting the concentration of Ca^2+^ and Si^4+^ ions at the nanotube surface and thereby, providing favorable sites for C-S-H nucleation.

In contrast, in the case of treated CNTs, the presence of hydroxyl and carboxyl groups enhances water adsorption, with the CNTs serving as adsorption sites for hydration products, water and ions [58,59]. In addition, these functional groups can chemically react with hydration products, thereby further promoting hydration [60].

### 2.2. Pore-Filling Effect and Composite Densification

The incorporation of CNTs also affects the poromechanical properties of the cementitious composite, as shown in Figure 5. At CNT concentrations below the CIC, both mesopores (4.5–25 nm) and micropores (<4.5 nm) are reduced, likely due to the physical filling of the pores by the nanotubes. However, as CNT content approaches the CIC, mesopores exhibit an opposite trend, with an increase in their fraction. This suggests that individual CNTs effectively fill the C-S-H gel pores, while higher concentrations promote bundling and agglomeration. Regarding entrapped air voids (25–100 μm), their volume fraction increases at all CNT concentrations, particularly around the CIC, although the overall porosity remains lower than that of the reference. The increase in air voids is attributed to the reduced fluidity of the mixture from CNT incorporation [29].

Nevertheless, the pore-filling effect of well-dispersed CNTs contributes to a denser and stiffer C-S-H network, thereby enhancing the composite’s mechanical reinforcement [29,40,53,61].

### 2.3. Interfacial Bonding Mechanisms Between CNTs and Cement Matrix

The impact of CNT functionalization on the mechanical performance of cementitious composites remains controversial [55]. While several studies report that functionalization enhances the strength of the composites [40,58,62,63], others have observed detrimental effects on mechanical properties [55,64,65,66].

Interfacial interactions between the functional groups of treated CNTs and the cement matrix have been reported to enhance the composite’s microstructure, as demonstrated in the study by Li et al. [40]. In particular, reactions between carboxyl groups on the CNT surface and the hydration products C-S-H and Ca(OH)_2_ can improve the interfacial bonding at the matrix–filler interface, thereby promoting more efficient load transfer. As a result, the incorporation of 0.5 wt% of MWCNTs was shown to enhance the compressive strength up to 19% and the flexural strength up to 25%. Kang et al. [58] also reported that acid-treated CNTs participate in reactions between their surface carboxyl groups and cement hydration products, leading to the densification and strengthening of the interface.

Similarly, the study conducted by Nasibulina et al. [62] reported an improvement in interfacial strength due to bond formation between functional groups on the CNT surface (-COOH and -OH) and the cement hydration products. These functional groups participate in ion exchange reactions, in which the proton is replaced by metal ions present in the cement matrix. Consequently, the incorporation of 0.03 wt% of functionalized few-walled CNTs (FWCNTs) resulted in compressive strength approximately twice that of the reference sample prepared without FWCNTs. A comparable mechanism was suggested by Merodio-Perea et al. [63], who, using molecular dynamics simulations, demonstrated the formation of electrostatic interactions between the negatively charged oxygen atoms of the CNT functional groups and the Ca^2+^ ions of the hydration products. In addition, CNT functionalization with carboxyl groups promotes the formation of a hydrogen bonding network that contributes to the adhesion between CNTs and hydration products.

According to Lavagna et al. [64], the improvement in compressive strength observed in cementitious composites containing acidic-treated CNTs is strongly related to the functionalization time. Although prolonged (over 90 min) sulfonitric acid treatment may partially damage the graphitic lattice of CNTs, the mechanical properties still improve due to enhanced dispersion and strong interfacial adhesion. However, excessive oxidation can lead to structural damage and breakage of the CNTs, reducing their reinforcing capabilities. In fact, a decline in mechanical properties was observed after 120 min of oxidation, indicating that the degradation of the graphitic structure becomes the dominant effect.

A case study reported by Musso et al. [65] indicated that the addition of functionalized CNTs to a cementitious matrix leads to a deterioration in both flexural and compressive strength. This weakening was attributed to the high hydrophilicity of functionalized CNTs, making them able to absorb most of the water and thereby hindering the proper hydration. This interpretation was supported by the relatively low amount of tobermorite gel formed during the hydration of calcium silicate phases, known to strongly influence the mechanical strength of cementitious materials [67]. In contrast, the use of pristine and annealed CNTs in the same study resulted in an improvement in the mechanical properties of the composite.

Similarly, Hawreen et al. [55] reported that the incorporation of carboxyl and hydroxyl functionalized CNTs resulted in a lower improvement in flexural and compressive strength compared with that of their unfunctionalized counterparts. The study also evidenced that ettringite was present in all cement pastes, but in higher amounts in those containing functionalized CNTs. The higher formation of ettringite may negatively affect the composite’s properties, in addition to the previously discussed factors such as structural defects induced by functionalization and possible reagglomeration. Analogous observations regarding the detrimental effect of expansive ettringite formation on the composite’s strength were also reported by Abu Al-Rub et al. [66].

The beneficial effects of untreated CVD-grown CNTs on the flexural strength and stress intensity factor (*K*_IC_) of cementitious composites were reported by Luo et al. [68]. The observed increases in mechanical properties were attributed to the superior bridging capability of well-dispersed nanotubes across the crack-opening zone.

Overall, the apparently contradictory effects of CNT functionalization on the mechanical performance of cementitious composites can be attributed to the competing mechanisms induced by surface treatment. On the one hand, the introduction of oxygen-containing functional groups can improve CNT wettability, dispersion and interfacial interaction with hydration products, potentially enhancing interfacial adhesion and load transfer. On the other hand, excessive oxidation may damage the graphitic structure of CNTs, while increased hydrophilicity may alter water availability and cement hydration. Changes in hydration product formation, including the increased presence of ettringite reported in some studies, may further affect the mechanical response. Therefore, functionalization should not be considered inherently beneficial or detrimental. Its effectiveness depends on the balance between interfacial improvement and possible adverse effects on CNT integrity and matrix hydration. This balance is also influenced by CNT dosage, nanotube characteristics, treatment conditions, dispersion procedure and mixture composition. Consequently, differences in experimental protocols should be considered when comparing the reported mechanical outcomes, and the benefits of functionalization should be evaluated in relation to the specific characteristics of each cementitious system. A comparison of experimental conditions and reported effects on the mechanical performance of cementitious composites is shown in Table 1.

### 2.4. Mechanisms of Crack Inhibition in CNT-Reinforced Cementitious Composites

Crack-bridging is, in fact, widely recognized as one of the main reinforcement mechanisms provided by CNTs in cementitious matrices, along with pull-out and crack deflection, which collectively contribute to the strength of the improved composite [56].

A strong interaction between CNTs and the cement matrix enables an effective crack-bridging mechanism. CNTs can stretch across the developing microcracks, facilitating load transfer between crack faces and thereby delaying crack propagation [19,69,70]. According to observations by Makar et al. [56], the typical widths of cracks bridged by CNTs are generally below 1 μm. By bridging the crack surfaces, CNTs can effectively connect two sides of fractured grains, thus preventing complete separation, as shown in Figure 6c.

At ultimate failure, two main mechanisms are commonly reported: CNT pull-out and CNT fracture, which depend on the strength of the filler–matrix interfacial adhesion [71]. Pull-out typically occurs when larger cracks induce the debonding and sliding of CNTs from the matrix, thereby indicating weak interfacial adhesion (Figure 6a,b). In contrast, CNT fracture is associated with stronger interfacial bonding, where CNTs remain anchored to the matrix and fail under load.

**Figure 6:**
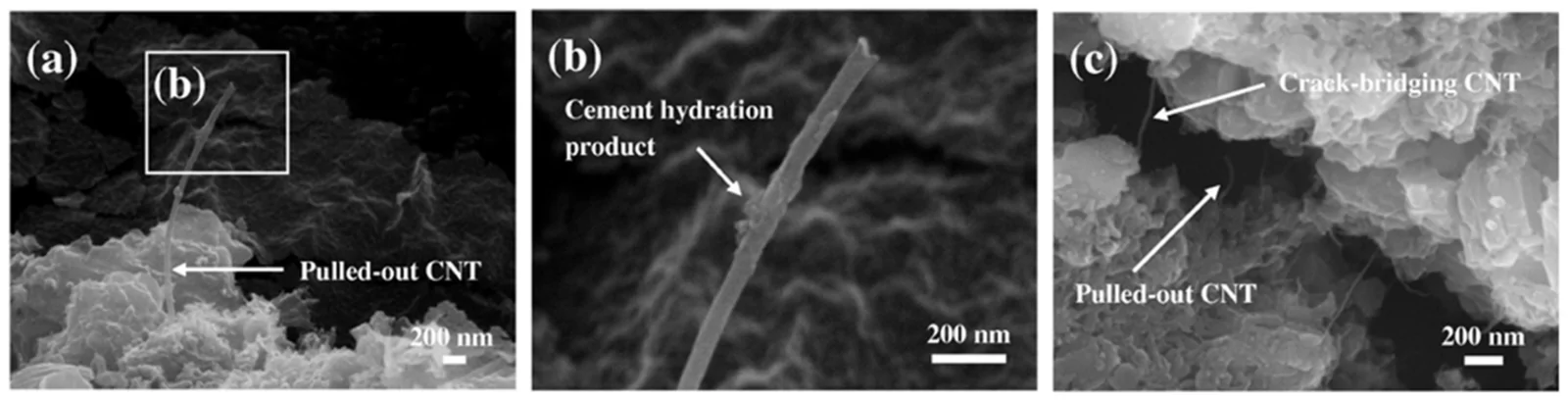
SEM images of CNT-reinforced cement composites showing (**a**), (**b**) CNT pull-out and (**c**) bridging (adapted from [72]).

Makar et al. [56] also suggested that CNT bundles may promote crack deflection, particularly in specimens with poor CNT dispersion when larger bundles are present. This interpretation was based on the observed adhesion of CNT bundles to the fracture surfaces of hydration products, which may indicate that propagating cracks deviated around the bundles rather than directly through them (Figure 7a).

Makar et al. [56] further proposed that CNT bundles may contribute to energy dissipation through the progressive separation of individual nanotubes within the bundle (Figure 7b). According to this interpretation, the gradual overcoming of the van der Waals interactions between adjacent CNTs during loading could absorb additional fracture energy.

The reinforcing mechanisms discussed above explain how CNTs influence the mechanical behavior of cementitious materials. However, the extent to which these mechanisms translate into measurable improvements in mechanical performance depends on several factors, including CNT dispersion, interfacial bonding, nanotube geometry, and defectiveness, as well as their interactions with the cementitious matrix. Predictive models, discussed in the following section, provide a framework for relating these factors to the resulting mechanical properties and for quantitatively assessing the effects of CNT incorporation. Establishing this link between the underlying reinforcement mechanisms and their representation in predictive models is essential for understanding the factors governing the mechanical performance of CNT-reinforced cementitious composites.

## 3. Models for Predicting Mechanical Properties

It is well established that CNTs have the potential to significantly enhance the mechanical properties of cementitious materials. While extensive experimental research has been conducted to investigate their influence on mechanical behavior, comparatively limited work has addressed their modeling [73].

This section aims to review the existing modeling approaches for estimating the mechanical properties of CNT-reinforced cementitious composites. These approaches encompass analytical formulations based on semi-empirical equations and numerical methods (such as the finite element method), which are well suited for capturing the microstructure of complex composite materials, as well as more recent machine learning techniques, validated against available experimental data. The above-mentioned modeling approaches are summarized in Figure 8.

### 3.1. Analytical Methods

Several theoretical models have been proposed in the literature to predict both the elastic modulus and flexural strength of composite materials. With regard to the estimation of the elastic modulus, the classical micromechanical models, such as Voigt and Reuss bounds, as well as more advanced formulations, including the Halpin–Tsai and Mori–Tanaka models, are commonly adopted.

Voigt [74] and Reuss [75] formulations, expressed as “laws of mixtures”, provide estimates of the Young’s and shear moduli of heterogeneous materials, with a good approximation of the experimental properties in the case of continuous fiber reinforcement. As demonstrated by Hill [76], these formulations represent the upper (Voigt) and lower (Reuss) bounds of the elastic properties of composite media. While both assume a homogeneous strain field, the Voigt model is appropriate for loading parallel to a fiber-shaped reinforcement, whereas the Reuss model provides a better approximation for configurations where the applied forces are perpendicular to the inclusion axis [73].

The Halpin–Tsai model provides a semi-empirical approach for estimating the properties of fiber-reinforced composite materials, accounting for both the volume fraction and the geometry of the reinforcing phase [77]. Based on a parallel spring analogy, the formulation considers fiber and matrix contributions only, neglecting interfacial interaction between them. The general form of the Halpin–Tsai relationship is given by Equation (1) [78]:

(1) $$\frac{P}{P_{m}}=\frac{1+\zeta \eta v_{f}}{1-\eta v_{f}}\;with\;\eta =\frac{\frac{P_{f}}{P_{m}}-1}{\frac{P_{f}}{P_{m}}+\zeta }$$

where *P* represents the property of the composite, *P_m_* and *P_f_* the corresponding matrix and fiber properties, *ζ* a parameter linked to the reinforcement geometry and *v_f_* the volume fraction of the fibers.

However, the conventional Halpin–Tsai model fails to accurately predict the mechanical properties of nanocomposites, as it neglects critical factors such as the influence of CNTs on the cement hydration process, their dispersion, and their bonding characteristics within the hosting matrix.

To address these limitations, the study conducted by Ramezani et al. [79] identifies the most influential experimental variables and incorporates their interactions affecting the mechanical properties, thereby enhancing the predictive capability of the base model for CNT-reinforced cement composites. The selected influential parameters include CNT concentration and/or volume fraction, aspect ratio, ultrasonication energy, superplasticizer-to-CNTs ratio, water-to-cement ratio, sand-to-cement ratio and hydration age. To this end, three critical factors are introduced:

- Dispersion factor *η_D_*, which provides a correlation between CNT dispersion and the aspect ratio, *AR*; volume fraction in aqueous solution, *v_f-w_*; superplasticizer-to-CNTs ratio, *SP*/*CNTs*; and ultrasonication energy indicator, *UEI* (a dimensionless index). It can be expressed as follows:

(2) $$\eta_{D}=1-\left[\frac{AR*v_{f-w}}{UEI*\left(\frac{SP}{CNTs}\right)}\right]\;with\;UEI={\left(t_{s}\right)}^{UA}$$

where *t_s_* is the duration of the ultrasonication process in minutes, and *UA* is the ultrasonication amplitude expressed as a percentage.

- Matrix factor, *η_M_*, considers both water-to-cement ratio, *w*/*c,* and sand-to-cement ratio, *s*/*c*. It can be expressed as:

(3) $$\eta_{M}=\frac{\left(1+s/c\right)}{\left(w/c\right)}$$

- Hydration age factor, *η_H_*, considers CNT concentration *κ*, expressed as weight percent of cement powder, and hydration age of cement *t*, expressed in days. It can be computed as follows:

(4) $$\eta_{H}={\left(\frac{t}{4+0.85t}\right)}^{\kappa }$$

According to Equation (5), these critical factors can be incorporated into the Halpin–Tsai model to predict the composite’s elastic modulus [80].

(5) $$E_{c}=\eta_{D}\eta_{M}\eta_{H}\frac{1+\xi E_{f^{\prime }}v_{f}}{1-E_{f^{\prime }}v_{f}}E_{m}\;with\;E_{f^{\prime }}=\frac{\chi_{0}\frac{E_{f}}{E_{m}}-1}{\chi_{0}\frac{E_{f}}{E_{m}}+\xi }$$

where *E_c_* represents the composite’s elastic modulus, *ξ*, the measure of fiber geometry (*ξ = 2L*/*d*), *E_f’_*, the equivalent elastic modulus of the fiber, *χ*_0_, the fiber orientation factor (equal to 1 for aligned fibers and equal to 3/8 and 1/6 for randomly orientated fibers in two and three dimensions, respectively), and *E_f_* and *E_m_*, the elastic moduli of fibers and matrix, respectively.

The probabilistic model proposed by Ramezani et al. [79] further refines Equation (5) by introducing calibrated exponents for the correction factors within a Bayesian framework. These exponents are identified through Bayesian inference, enabling the relative influence of dispersion, matrix characteristics, hydration, and other governing factors to be quantified, while accounting for uncertainties in the experimental database. The resulting model predicts the elastic modulus with an error of approximately 18 ± 1%, with respect to experimental data.

Another widely employed homogenization model for estimating the elastic properties of composite materials is the Mori–Tanaka model. This method, based on the Eshelby’s solution, accounts for the interactions of ellipsoidal inclusions by placing each inclusion in a stress field corresponding to the average stress field in the matrix [81]. The primary assumption of the Mori–Tanaka model is that the average strains in the inclusions and the matrix are related through a fourth-order tensor [82].

Benveniste [83], based on the Mori–Tanaka theory, developed a simplified expression for predicting the elastic modulus of composite materials. This method is limited to an anisotropic elastic two-phase composite, with the inclusion consisting of ellipsoidal particles which may be either aligned or randomly oriented. According to this model, the effective stiffness tensor ***C*** of the CNT composites can be expressed by means of Equation (6) [84]:

(6) $$C=\frac{\left(1-v_{f}\right)C_{m}+v_{f}C_{f}A}{\left(1-v_{f}\right)I+v_{f}A}$$

where ***C_m_*** and ***C_f_*** are the stiffness tensors of the matrix and fibers, respectively, ***A*** is the localization tensor of the average strain of inclusions, and ***I*** is the unit tensor of the fourth rank.

Falara et al. [85] compared the experimentally measured Young’s modulus of cement paste (*w*/*c* = 0.30) reinforced with 0.048 and 0.08 wt% MWCNTs with the values predicted by the Voigt, Reuss, and Benveniste–Mori–Tanaka models. As shown in Figure 9, among the three micromechanical models considered, the Benveniste–Mori–Tanaka model provides the closest agreement with the experimental results, whereas the Voigt and Reuss models significantly overestimate and underestimate the elastic modulus, respectively.

Besides elastic modulus, an additional frequently investigated mechanical property is flexural strength. In this context, the Kelly–Tyson model [86], originally developed to predict the tensile strength of composite materials, has also been applied to estimate the flexural strength of cementitious matrix composites [87,88]. Assuming equal elastic strain in both the fibers and the matrix, the tensile strength of the aligned continuous fiber-reinforced composites can be expressed in the form of the rule of mixtures using Equation (7) [86]:

(7) $$\sigma_{c}=\sigma_{f}v_{f}+\sigma_{m}\left(1-v_{f}\right)$$

where *σ_c_*, *σ_f_* and *σ_m_* are the tensile strength of the composite, fiber, and matrix, respectively.

When the fracture strain of the matrix is greater than that of the fiber, the ultimate tensile strength of the composite *σ_ult c_* is given by Equation (8) [86]:

(8) $$\sigma_{ult\;c}=\sigma_{ult\;f}v_{f}+{\sigma^{\prime }}_{m}\left(1-v_{f}\right)$$

where *σ_ult f_* is the fiber maximum stress before failure, and *σ’_m_* the stress on the matrix when the fibers reach their ultimate tensile stress.

By contrast, if the fiber volume fraction is lower than the critical value $v_{f}^{\prime }$, defined by Equation (9) as

(9) $${v\prime }_{f}=\frac{\sigma_{ult\;m}-{\sigma \prime }_{m}}{\sigma_{ult\;f}+\sigma_{ult\;m}-{\sigma \prime }_{m}}$$

where *σ_ult m_* is the matrix maximum stress before failure, the matrix can sustain the applied load after fiber fracture without the contribution of the fibers.

Consequently, the composite tensile strength is given by Equation (10).

(10) $$\sigma_{ult\;c}=\sigma_{ult\;m}\left(1-v_{f}\right)$$

To account for the contribution of semi-aligned short discrete fibers, Equation (8) was modified by Krenchel [89] by adding the effect of fiber length *L* and the orientation factor *χ*_0_ (equal to one for aligned fibers and equal to 3/8 and 1/6 for randomly oriented fibers in two and three dimensions, respectively). Therefore, the tensile strength of the composites can be calculated applying Equation (11).

(11) $$\sigma_{c}=\chi_{0}\left[\sum_{L=0}^{L<L_{c}}\tau_{i}\left(\frac{L}{d}\right)+\sum_{L=L_{c}}^{L}\left(1-\frac{L_{c}}{2L}\right)\sigma_{f}\right]v_{f}+\sigma_{m}\left(1-v_{f}\right)$$

where *L_c_* is the fiber critical length, *d* is the fiber diameter, and *τ_i_* is the interfacial bond stress.

Ramezani et al. [90] proposed another modified formulation of the Kelly–Tyson model, accounting for the fact that CNT lengths are typically below the critical length and that their volume fraction in cementitious composites is very low. The incorporation of these considerations in the model yields the following expression for the calculation of the composite’s flexural strength *σ_c_*, as expressed in Equation (12).

(12) σc=16τiARvf23+σm(1−vf23)

To account for the mechanisms of CNTs in cementitious materials, namely interfacial bonding, dispersion, and their influence on hydration process, and to capture their interactions, three critical factors (dispersion *η_D_*, matrix *η_M_* and hydration age *η_H_*) can be incorporated into the flexural strength expression, as defined in Equation (13).

(13) σc=ηDηHηM16τiARvf23+σm(1−vf23)

This expression is again formulated in a probabilistic manner using a Bayesian methodology to calibrate the model parameters, thereby improving the model’s predictive capability. The proposed probabilistic model is able to predict the flexural strength with a no greater than a 15% error rate relative to the experimental data.

The interactions among the influential parameters affecting the mechanical properties of CNT-reinforced cement composites have also been addressed by Hawreen et al. [55]. In their study, an equation is proposed to correlate the flexural strength of cement pastes containing CNTs with their aspect ratio and volume fraction. Assuming ideal dispersion and considering CNT pullout as the predominant failure mechanism, CNTs are assumed to provide a maximum load transfer capacity *F_CNT_* as expressed in Equation (14).

(14) FCNT=K∗σb∗fb∗AR13∗vf23

where *K* is a constant, *σ_b_* the bonding strength between the CNTs and the cement matrix, and *f_b_* is a factor related to the effective anchorage length of the CNTs.

### 3.2. Numerical Modeling Methods

The rising need for composite materials characterized by variable inclusion fractions and complex geometries has made numerical modeling a crucial tool for nanocomposite characterization [73]. The most widely used numerical approaches are molecular dynamics (MD) and the finite element method (FEM). MD simulations use numerical methods to model the time-dependent behavior of atoms and molecules in materials, while FEM predicts the mechanical response of materials by discretizing the domain into finite elements and solving equations to evaluate their interactions and overall behavior.

Multiscale modeling is commonly employed to investigate CNT–matrix interactions at the nanoscale and their effects on macroscopic behavior. The connection between the two scales is established via the representative volume element (RVE) concept. This approach enables the association at the macroscale of each material point with a corresponding RVE, which is designed to be sufficiently large to capture the material’s heterogeneity, while still remaining small relative to the overall dimensions of the structure [91].

Early on, the RVE technique was employed by Chan and Andrawes [92] to investigate the interaction between CNTs and a cement matrix. In this study, a finite element model was developed to simulate the pull-out behavior of a single CNT embedded in a cementitious matrix. The applicability of this modeling approach was previously demonstrated by Liu and Chen [93].

More specifically, ANSYS software was used to model the three fundamental components of the composite, namely the embedded CNT (Figure 10a), the CNT–matrix interface (Figure 10b) and the surrounding cement matrix (Figure 10c). The developed RVE pull-out model allowed for the investigation of several parameters, including interfacial shear strength, allowable slip, CNT Young’s modulus, residual stress and aspect ratio, as well as their interactions. The mechanical properties investigated were cement composite ductility and strength.

The simulation results indicated that the CNT–cement composite exhibiting high ductility requires a CNT with a relatively low elastic modulus (i.e., less than 500 GPa) and a strong interfacial bond with the cement matrix. Conversely, composites intended for structural applications characterized by high strength can be achieved using high-modulus CNTs, again characterized by high interfacial interaction, while their aspect ratio was found to have a negligible influence.

Unlike simplified CNT representations, the study conducted by Awol et al. [91] incorporates several CNT features into the developed model, investigating different approaches for SWCNT-reinforced cementitious composites to identify the factors influencing their Young’s modulus. More precisely, several three-dimensional RVEs were developed, as shown in Figure 11, including aligned CNTs, randomly oriented CNTs, randomly oriented curved CNTs, randomly oriented CNTs with agglomeration, randomly oriented CNTs with both agglomeration and curvature, and randomly oriented curved CNTs with agglomeration and voids.

The prediction of the composite’s Young’s modulus was carried out using an FEM simulation in Abaqus CAE software. The results, reported in Table 2, indicate that the model incorporating curved CNTs with agglomeration and voids provides the best agreement with the experimental data.

Furthermore, a parameter-sensitivity analysis, based on the Mori–Tanaka Mean Field homogenization method, was performed to identify the most influential parameters on composite performance, including agglomeration, CNT aspect ratio, volume fraction, CNT curvature, Young’s moduli of both the CNTs and the cement matrix, interfacial properties, CNT orientation and void content.

The study identified CNT curvature, agglomeration, and void content as the primary factors governing the stiffness of CNT-reinforced cementitious composites.

In the study conducted by Kavvadias et al. [94], the mechanical properties of MWCNT-reinforced cement composites were investigated using the random sequential adsorption algorithm, developed to generate non-overlapping matrix-inclusion three dimensional RVEs, as illustrated in Figure 12. FEM was performed using ANSYS software to evaluate Young’s modulus, as well as compressive and tensile stress.

Regarding the evaluation of Young’s modulus, Figure 13 presents a comparison between numerical simulation results, experimental data and predictions obtained from the micromechanical Halpin–Tsai model. It can be observed that both the numerical simulation and micromechanical model underestimate Young’s modulus for MWCNT contents of 0.08, 0.1 and 0.2 wt%. For a concentration of 0.3 wt%, the experimental value is lower than that at 0.2 wt%, which can be attributed to inadequate CNT dispersion in the matrix, an aspect which is not captured by the numerical model.

Furthermore, the tensile and compressive strengths predicted by numerical simulations are shown in Figure 14 and compared to the experimental results. For MWCNT concentrations below 0.2 wt%, the numerical predictions are in good agreement with the experimental data. However, for higher CNT contents, the model fails to accurately account for agglomeration and bundle formation, which promote crack initiation and lead to reductions in both tensile and compressive strengths.

Regarding the flexural behavior of CNT-reinforced cement composites, it is worth mentioning the work conducted by Anastopoulos et al. [73], in which both the random orientation tensor algorithm and the periodic geometry algorithm were employed to construct RVEs with different MWCNT volume fractions (0.6% and 1.2%). The finite element method implemented in ANSA software was used to simulate flexural tests on pre-cracked specimens, and the results were subsequently validated against experimental data.

Different input parameters were considered, including Poisson’s ratio of the reinforcement and its effective elastic modulus, in order to predict the flexural behavior of the cementitious composite. The results showed that both effective elastic modulus and Poisson’s ratio significantly influence the composite’s flexural response, with deviations up to 13% from the experimental results.

The best agreement with experimental data was obtained for the CNT effective elastic modulus of 470 GPa and a Poisson’s ratio of 0.10 for both investigated CNT volume fractions.

### 3.3. Machine-Learning-Based Models

Conventional numerical approaches are often computationally prohibitive for real-world applications involving macroscopic components subjected to non-uniform, multiaxial strain fields, as they require the explicit resolution of micron-scale RVEs [95]. At the same time, traditional experimental characterization of composite materials is increasingly constrained by its significant demands in both time and labor.

In this context, machine learning (ML) has emerged as a promising alternative; however, its reliability strongly depends on data quality, dataset size, and feature selection. By leveraging large experimental and simulation datasets, ML models enable accurate predictions of composite material performance while significantly reducing computational costs related to conventional physics-based methods. Furthermore, ML facilitates multiscale modeling by linking atomistics simulation to macroscopic behavior, thereby bridging the gap between microstructural characteristics and bulk properties [96].

Recently, increasing attention has been devoted to the application of ML techniques for predicting the mechanical properties of CNT-reinforced cementitious composites. Among the early contributions, Huang et al. [97] employed artificial neural network (ANN) and support vector machine (SVM) models to predict both compressive and flexural strength.

Among ML approaches, ANN models are widely adopted due to their capability to capture nonlinear relationships, although their performance is often constrained by limited datasets and risk of overfitting. A typical ANN architecture (Figure 15) consists of input, hidden, and output layers, each comprising multiple interconnecting neurons. The input layer receives and delivers input variables, the hidden layer performs intermediate computations, and the output layer generates the final predictions. Activation functions, implemented in the hidden and output layers, introduce nonlinearity, enabling the model to capture intricate relationships within the data [96].

In contrast, SVM relies on nonlinear mapping of input data in a high-dimensional feature space through transformation functions. Predictions are then obtained using kernel functions, including linear, radial basis function (RBF), polynomial, sigmoid and exponential RBF [96].

In the study conducted by Huang et al. [97], nine input variables are considered, including cement type, *w*/*c* ratio, CNT content, external diameter and length, functionalization method, curing time and temperature, and dispersion method. While ANN showed superior performance for relatively small datasets, SVM with an RBF kernel proved more suitable for larger datasets. However, the comparison between models across different studies remains challenging due to the lack of standardized datasets and evaluation metrics. The best ANN performance was achieved using the Tanh activation function and an Adadelta optimizer, with predictions in good agreement with the experimental data, as shown in Figure 16. Minor discrepancies between predicted and measured values were attributed to unaccounted parameters affecting model accuracy.

A more comprehensive dataset was collected by Adel et al. [98], incorporating ten input variables, including *w*/*c* ratio, CNT type, content, length and diameter, *s*/*c* ratio, surfactant type, dispersion method, curing time, and the compressive or flexural strength of the control sample. In this study, four ensemble-learning algorithms, namely random forest (RF), adaptive boosting (AdaBoost), gradient boosting (GB) and extreme gradient boosting (XGB), were evaluated alongside a standalone decision tree (DT) model to predict the mechanical properties of CNT-reinforced cementitious pastes and mortars. The dataset was randomly divided into training and testing subsets in an 80:20 ratio, and the predictive performance of the models was assessed by comparing the predicted and experimental values for the test dataset.

The DT is a widely used ML method composed of a root node, leaf nodes and branches, as illustrated in Figure 17, where the root node represents the input data and leaf nodes the predicted outputs [99].

RF, one of the most commonly used ensemble learning methods for regression, constructs a set of multiple DTs to improve robustness and accuracy. AdaBoost employs an iterative boosting strategy emphasizing misclassified instances to enhance model performance. GB similarly relies on an iterative procedure, minimizing a cost function by sequentially correcting the errors of previous models, whereas XGB represent a scalable and optimized implementation of this approach [96,98].

The predictive capability of the models was evaluated using scatter plots comparing the predicted and experimental values for the test dataset. Among the investigated algorithms, XGBoost demonstrated the best performance, exhibiting the highest agreement with the experimental data for both compressive (Figure 18) and flexural strength (Figure 19) [98].

In the study by Bagherzadeh et al. [100], two ensemble ML algorithms, RF and GB, were implemented using the SciKit-learn library in Python to predict the compressive and flexural strength of CNT-reinforced cementitious composites. The models considered multiple input parameters, including cement type, *w*/*c* ratio, content of CNTs, external diameter, length, functionalization method, curing days and temperature, and dispersion method. Figure 20 shows how the predicted values of both compressive and flexural strength are in good agreement, with only minor discrepancies attributable to unaccounted variables affecting model accuracy. Both algorithms demonstrated higher predictive accuracy for compressive strength than for flexural strength, with GB outperforming RF overall. Sensitivity analysis further revealed that the external diameter of the CNTs was the most influential parameter for predicting flexural strength, whereas the *w*/*c* ratio had the greatest impact on the prediction of compressive strength.

Li et al. [101] investigated the prediction of compressive strength in cement paste, mortar and concrete using multiple ML models, including linear regression (LR), support vector regression (SVR), RF and XGB. LR, one of the most fundamental ML algorithms, relies on the least-square method to establish a linear relationship between input variables and target response. In contrast, SVR is based on the principle of SVM and introduces a tolerance margin *ε* within which prediction errors are not penalized.

The models were trained using 11, 12 and 13 input variables for cement paste, mortar and concrete, respectively. The results demonstrated satisfactory predictive performance for both RF and XGB, with the latter achieving the highest coefficient of determination *R*^2^ equal to 0.93.

To enhance model interpretability, shapely additive explanations (SHAP) were employed, providing insights into the contributions of individual features to predictions. The analysis revealed that CNT length, diameter and content demonstrate a significant influence on compressive strength, whereas surfactant type plays a comparatively minor role. Furthermore, SHAP analysis identified optimal values of approximately 20 μm for CNT length, 25 nm for diameter and 0.1% for content.

Compared with previous studies, the work conducted by Yang et al. [102] introduces, for the first time, the effect of specimen size into ML modeling. Specifically, three ML approaches, namely RF, ANN and AutoGluon-Tabular (AGT), were employed to predict the 28-day compressive strength of CNT-reinforced cementitious composites. The AGT approach, an automatic ML method, integrates multiple algorithms and enables efficient model development with minimal coding effort.

The models were trained using 17 input features, and, unlike in previous studies, a Gaussian function was adopted to represent the distribution of CNT dimensions rather than fixed values. The results showed that ANN achieved the highest predictive accuracy among the models considered, whereas the AGT demonstrated greater efficiency and ease of implementation in handling the dataset.

With regard to the prediction of elastic modulus, the study by Okasha et al. [103] provides relevant insights. Specifically, the interactions among seven key input variables were investigated using three ML models, i.e., ANN, SVR and histogram gradient boosting (HGB). HGB is a DT-based algorithm that combines GB with histograms to efficiently handle numerical features.

Among the considered models, ANN achieved the highest accuracy in predicting the elastic modulus, as indicated by lower roots mean square error (RMSE) and mean absolute error (MAE) values. Conversely, the HGB model showed superior performance in estimating the cementitious composite’s flexural strength.

Abbas et al. [104] introduced a deep learning (DL) model specifically developed to predict both the compressive and flexural strength of cementitious composites reinforced with CNTs. DL, often referred to as a deep neural network (DNN), is based on ANN with multiple hidden layers, enabling the effective modeling of complex and nonlinear relationships. As depicted in Figure 21, while conventional ANN introduces only a single hidden layer, DL comprises multiple layers with numerous interconnected neurons, allowing a more efficient representation of complex data structures [105].

The model developed by Abbas et al. [104] was trained using 14 input variables, and the results, reported in Figure 22 for compressive strength and Figure 23 for flexural strength, demonstrated high accuracy on the training dataset, as indicated by the close agreement between predicted and actual values. However, a noticeable reduction in accuracy was observed when the model was applied to unseen data, suggesting some limitations in generalization performance.

More recently, Ramezani et al. [106] investigated the prediction of the elastic modulus and flexural strength of CNT-reinforced cementitious materials by analyzing a comprehensive set of 19 input variables and employing multiple artificial intelligence (AI)-based models, including DNN, SVR and ensemble-bagging. Bagging, also known as bootstrap aggregation, is a two-step ensemble learning technique that involves training multiple base learners, typically DTs, on subsets of the original training dataset, followed by aggregation of the predictions, usually through averaging to improve robustness [107,108].

The results indicated that the DNN model achieved the highest predictive accuracy, at the expense of increased computational cost. In contrast, SVR and bagging approaches were more computationally efficient but less effective in capturing complex nonlinear relationships. Model performance was evaluated in terms of mean absolute error (MAE), mean squared error (MSE), root mean squared error (RMSE), mean absolute percentage error (MAPE) and the coefficient of determination (R^2^).

A comparative summary of the input and output parameters and the performance metrics, reported by Ramezani et al. [106] and the previously discussed study for predicting flexural strength, is presented in Table 3 and Table 4. It should be noted that the reported performance metrics are specific to the datasets, input variables, data preprocessing procedures and validation strategies adopted in each study. Consequently, these values do not support a direct quantitative comparison or ranking of model performance across different studies. Rather, they provide an overview of the predictive performance reported under different experimental and modeling conditions.

Overall, despite their promising predictive capabilities, ML models in this field are still limited by data scarcity, variability in experimental conditions, and poor generalization outside the training domain.

### 3.4. Comparative Assessment of Predictive Modeling Approaches

Traditionally, the mechanical properties of composites have been determined through experimental testing, including tensile, compressive, and flexural tests. Although experimental characterization provides the most reliable assessment of material behavior, it is often laborious, time-consuming, and costly due to the required equipment and specimen preparation. Moreover, the measured properties may be affected by experimental uncertainties arising from testing procedures, equipment limitations, and material variability [99].

To overcome these limitations, analytical, numerical, and data-driven approaches have been increasingly adopted to predict the mechanical behavior of CNT-reinforced cementitious composites. More precisely, analytical and semi-empirical models are computationally efficient and provide valuable physical insight into the governing reinforcement mechanisms. However, their predictive capability is constrained by simplifying assumptions that cannot fully represent the heterogeneous nature of cementitious nanocomposites.

Numerical methods, such as finite element modeling, provide a more realistic description of microstructural features, but they are computationally demanding and typically require extensive calibration and validation against experimental data.

In recent years, artificial intelligence, particularly machine learning, has emerged as a powerful alternative for predicting the mechanical properties of cementitious composites. By learning complex nonlinear relationships directly from experimental data, ML models can achieve high predictive accuracy while significantly reducing computational time. Nevertheless, their performance strongly depends on the availability, quality, and representativeness of the training dataset, and they generally lack the physical interpretability of conventional mechanics-based models [109]. Consequently, current research is increasingly focused on hybrid modeling strategies that combine physics-based formulations with machine learning algorithms, aiming to integrate physical consistency, predictive accuracy, and computational efficiency [110].

Despite these advantages, several limitations must be considered when applying ML to CNT-reinforced cementitious composites. First, data quality and availability are critical challenges [111,112]. Experimental datasets may be limited in size and heterogeneous in terms of CNT type, dosage, functionalization, dispersion procedure, water-to-cement ratio, curing conditions and testing protocols. Measurement uncertainty and missing experimental parameters can reduce the reliability of model predictions. Furthermore, when data are collected from multiple studies, differences in experimental procedures and material formulation may introduce variability that cannot be fully explained by the input variables included in the model.

Second, appropriate model validation is essential to assess predictive reliability and to ensure generalizability. Hold-out validations and cross-validation are commonly used strategies, but their suitability depends on the dataset size, structure and intended application of the model. In the hold-out approach, the available data are divided into two subsets, namely training and testing sets, which are used to fit the model and evaluate its predictive performance, respectively. However, when datasets are small, this division may result in insufficient or unrepresentative subsets, leading to uncertain performance estimates. *K*-fold cross-validation addresses this limitation by repeatedly training and evaluating the model across different data partitions, thereby providing a more stable estimate of predictive performance when data are limited. Nevertheless, cross-validation does not eliminate the risk associated with small datasets, and its results depend on how data are partitioned. Moreover, hyperparameter tuning should be performed without using a final test set to avoid optimistic estimates of model performance [112,113].

Third, overfitting represents a significant risk, particularly when the number of experimental observations is small related to the complexity of the model. Overfitting arises when the model adjusts its many degrees of freedom to the properties of the training dataset, learning noise or dataset-specific patterns rather than relationships that generalize to unseen observations. To avoid this risk, validation data should not be used in training but used instead to assess the error in the model only. However, this measure cannot compensate for insufficient or unrepresentative training data [113,114].

Finally, the generalizability of ML models remains a major challenge. A model trained on a limited experimental dataset size may perform well under similar conditions but fail when applied to previously unseen experimental settings [115]. This limitation is particularly relevant to CNT-reinforced cementitious composites, in which variation in CNT characteristics, dispersion procedures, mixture composition and curing conditions may affect the relationships learned by the model. Therefore, predictive accuracy should be assessed not only through conventional error metrics but also through validation on datasets representing different material systems and experimental conditions.

## 4. Challenges and Perspectives for Engineering-Scale Implementation

Despite the promising mechanical improvements reported for laboratory-scale CNT-reinforced cementitious composites, their upscaling remains challenging. A major issue is the achievement of uniform CNT dispersion in large batches, as conventional mixing procedures may not provide equivalent dispersion efficiency when applied to larger volumes [116]. Although ultrasonication is widely employed in laboratory studies, its engineering-scale implementation may involve substantial energy demand and is limited due to constrains imposed by equipment [117]. Moreover, excessive sonication energy or prolonged treatment may damage CNT structure, potentially compromising reinforcement efficiency [118]. High-shear mixing and suitable dispersants may represent alternative strategies, although their effectiveness, energy requirements and compatibility with cementitious systems require further validation [119].

Workability is another important consideration, as CNT incorporation and the use of dispersants may alter the rheological properties of fresh mixtures, consequently affecting flowability [120,121]. Furthermore, the economic feasibility of CNT-reinforced cementitious composites depends on several factors, including CNT production costs, dosage and the energy required for mixing and dispersion.

Reproducibility is also essential, since variations in CNT characteristics, dispersion quality, mixing equipment, operating conditions and batch size may lead to inconsistent mechanical properties. Therefore, future research should prioritize the development of standardized processing and characterization protocols to improve reproducibility and enable reliable comparisons across studies [122].

Addressing these challenges is essential to ensure reproducible performance and facilitate the transition from laboratory research to practical engineering applications.

## 5. Conclusions

Cement-based materials remain indispensable in the construction industry owing to their favorable strength, stiffness, durability and widespread availability. Nevertheless, their intrinsic brittleness continues to limit the development of materials with enhanced resistance to cracking and fracture. In this context, this review has examined the potential of CNTs as reinforcing agents in cementitious matrices for improving mechanical performance. Based on the analyzed literature, several key findings can be highlighted:

- CNTs have been consistently identified as effective nucleation sites, although their efficiency strongly depends on dispersion quality and functionalization.
- CNT incorporation contributes to pore refinement and microstructural densification, enhancing the compactness and continuity of the C-S-H network.
- The role of CNT functionalization in governing interfacial interactions with the cementitious matrix has been assessed, revealing both beneficial and adverse effects on mechanical properties.
- CNTs act as effective crack inhibitors through mechanisms such as crack-bridging, pull-out and crack deflection, which collectively enhance toughness and resistance to fracture.

This work also reviewed current modeling approaches for predicting mechanical properties of CNT-reinforced cementitious composites. Analytical models, while useful for estimating elastic modulus and flexural strength, are often limited by their inability to fully capture the complex interactions governing composite behavior. Numerical approaches, particularly those based on RVEs and implemented via FEM, provide improved insights into these interactions. More recently, machine learning and artificial intelligence techniques, including ANN, SVM, DT, RF and DL, have demonstrated strong potential in capturing nonlinear relationships between microstructural features and macroscopic properties.

Overall, this review consolidates the current state of knowledge regarding the use of CNTs as reinforcements in cementitious materials. However, significant challenges remain, particularly in achieving uniform CNT dispersion, ensuring scalability, and integrating physics-based and data-driven modeling approaches. Addressing these issues will be crucial for the practical implementation of CNT-reinforced cementitious composites.

## Figures and Tables

**Figure 1:**
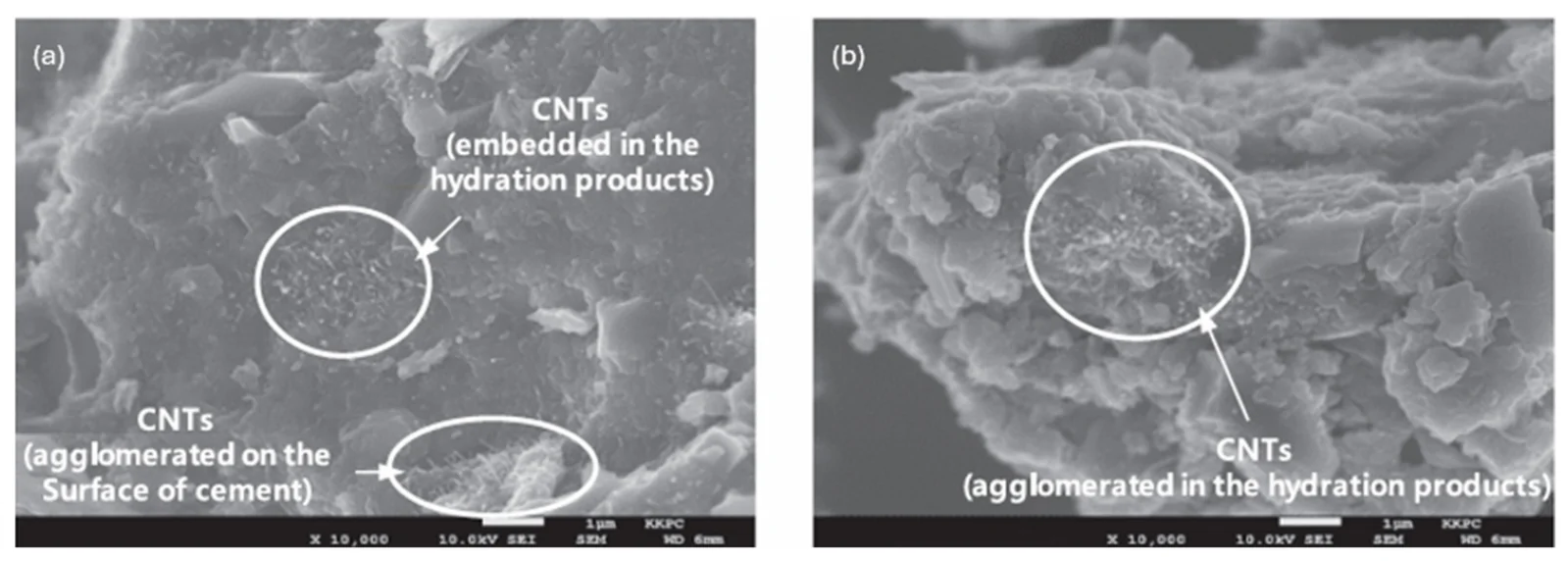
SEM images of the OPC/CNT composite at (**a**) 0.8 wt% and (**b**) 1.0 wt% concentrations [20].

**Figure 2:**
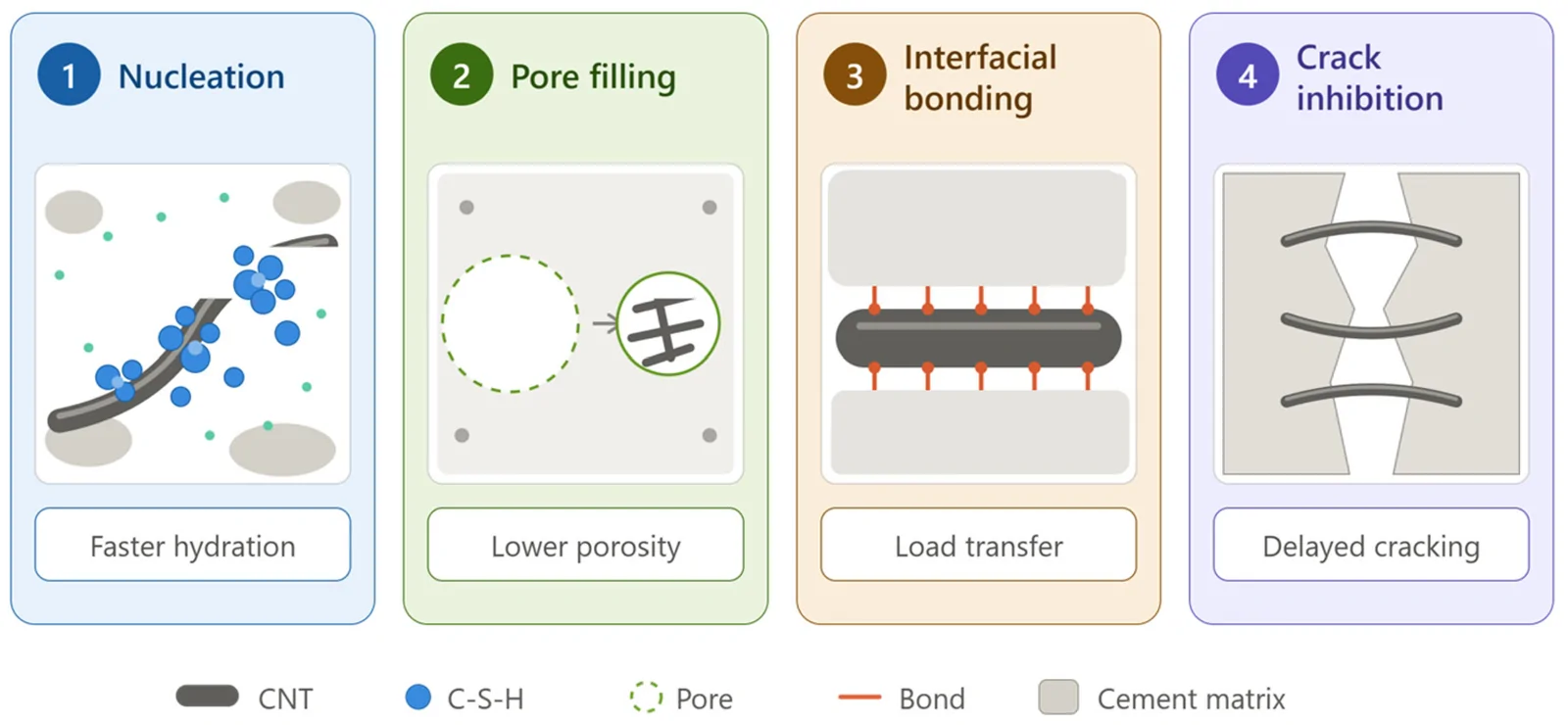
CNT reinforcement mechanisms in cementitious composites.

**Figure 3:**
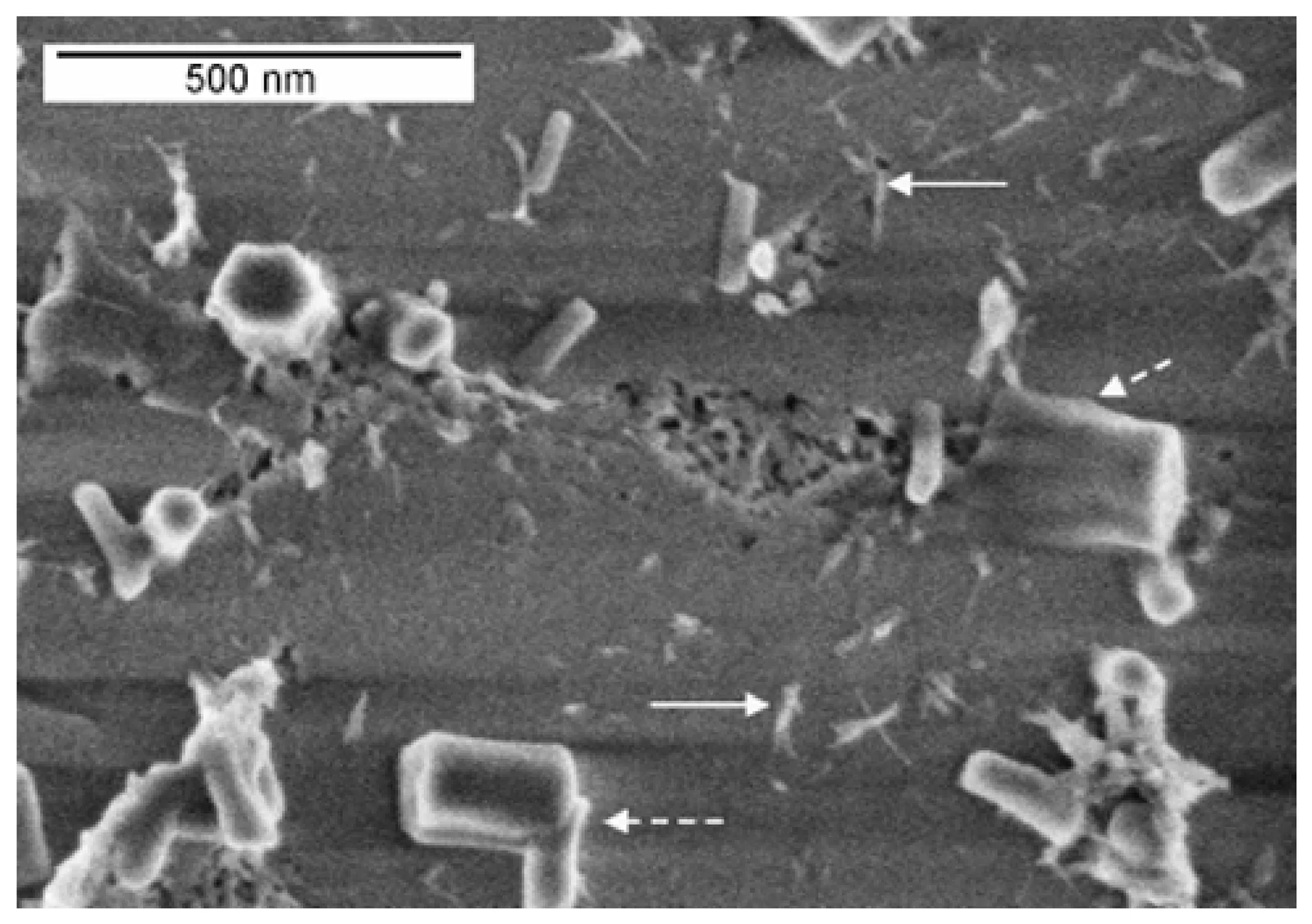
OPC sonicated without nanotubes after 135 min of hydration. Solid arrows indicate C-S-H, while dashed arrows point to C_3_A hydration products formed prior to the induction period [54].

**Figure 4:**
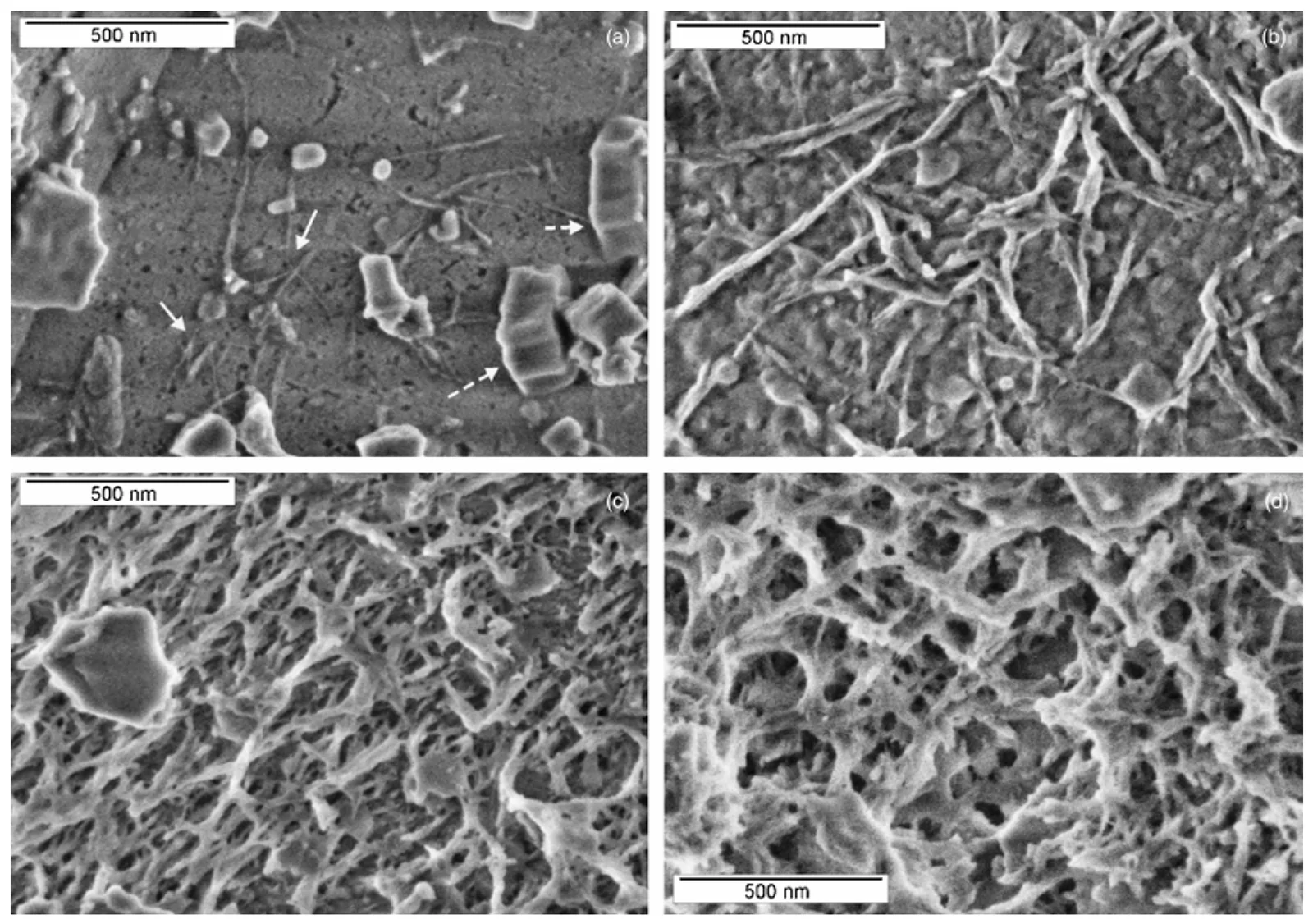
(**a**) Intersecting SWCNT bundles (solid arrows) and early prismatic hydration products (dashed arrows) after 60 min of hydration. (**b**) Growth of C-S-H on SWCNT bundles after 135 min of hydration. (**c**) C-S-H meshwork on grain surface after 180 min of hydration. (**d**) Evolution of porous structure of hydration products after 240 min of hydration [54].

**Figure 5:**
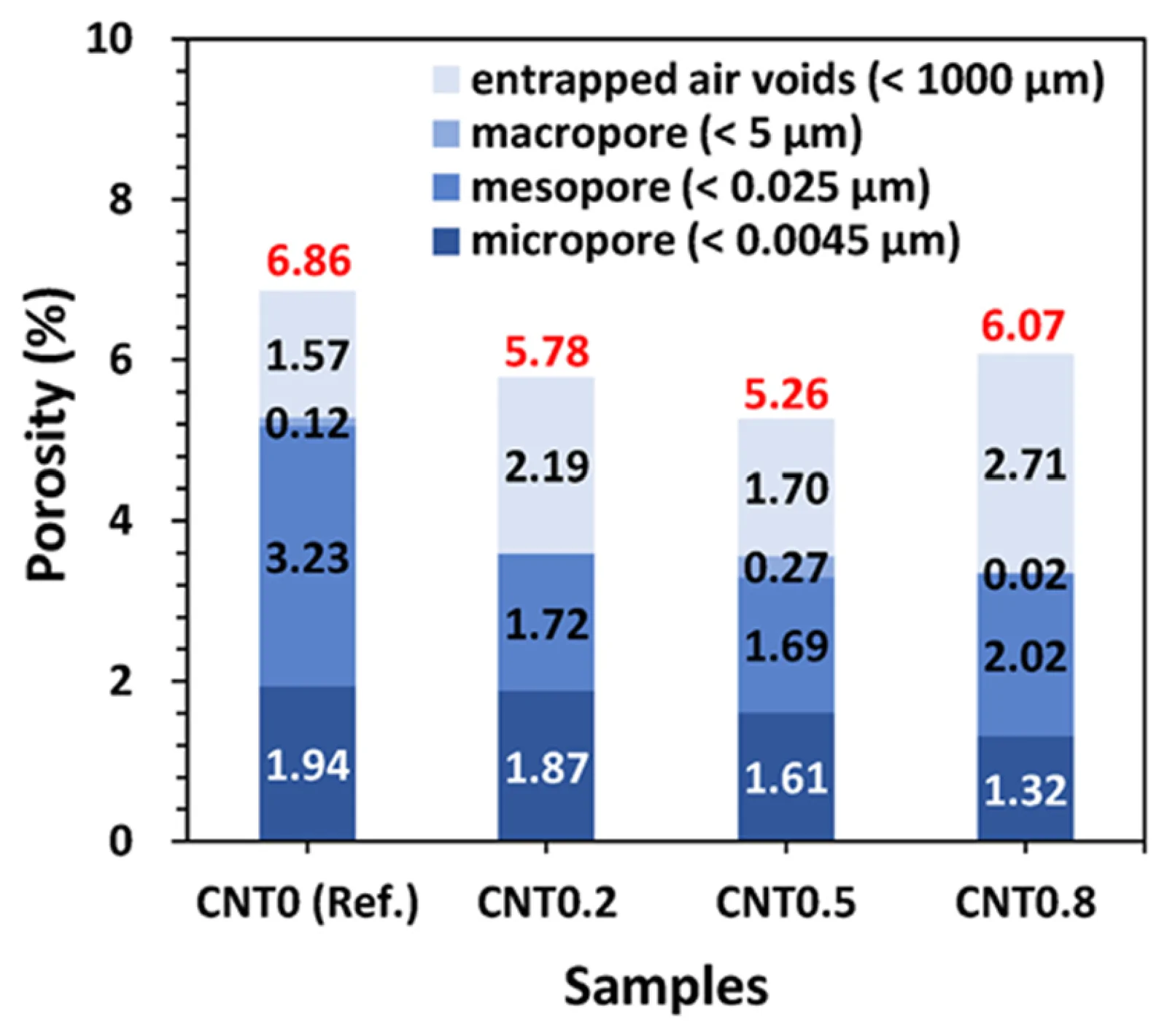
Pore volume distribution and total porosity (red numbers on top of the bars) after 28 days for different CNT concentrations [29].

**Figure 7:**
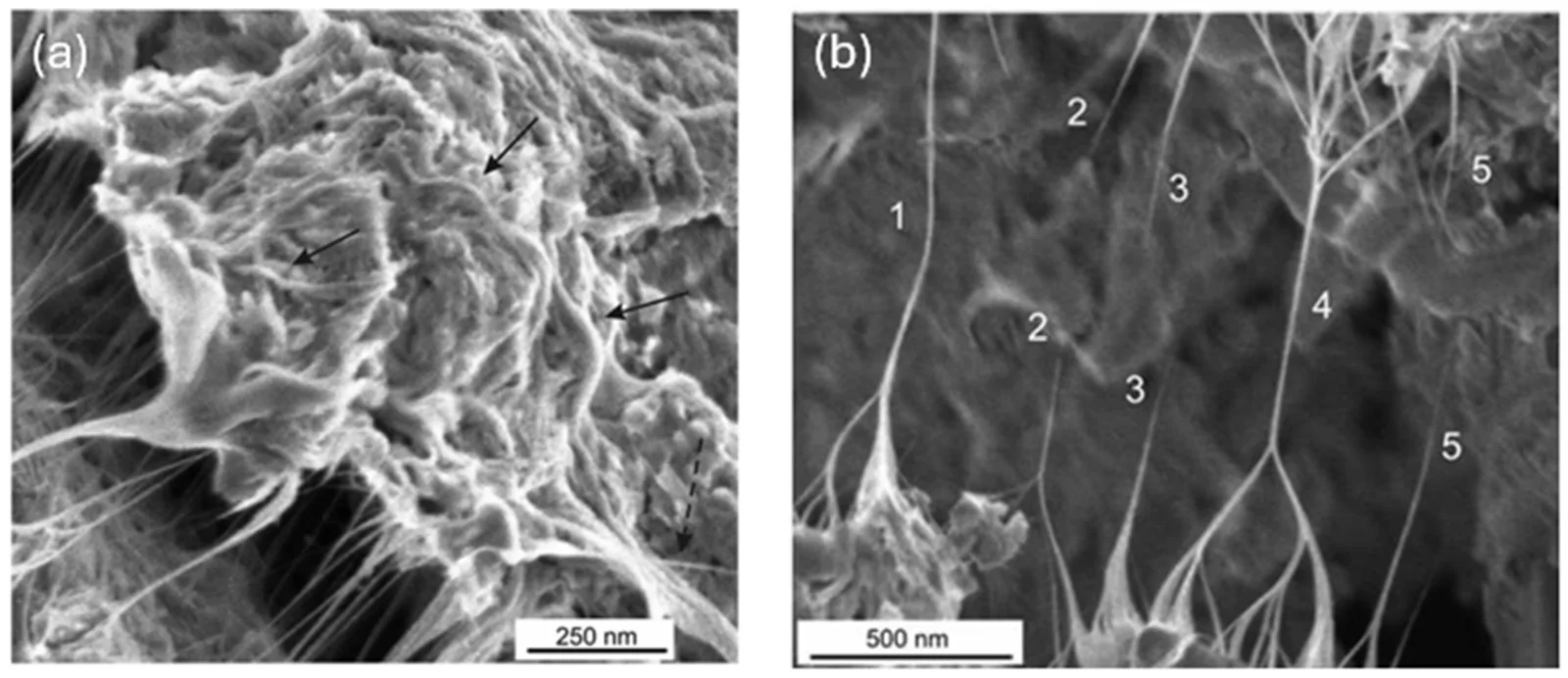
(**a**) Crack deflection (solid arrows) and fiber pull-out (dashed arrow). (**b**) Separation of CNT bundles [56].

**Figure 8:**
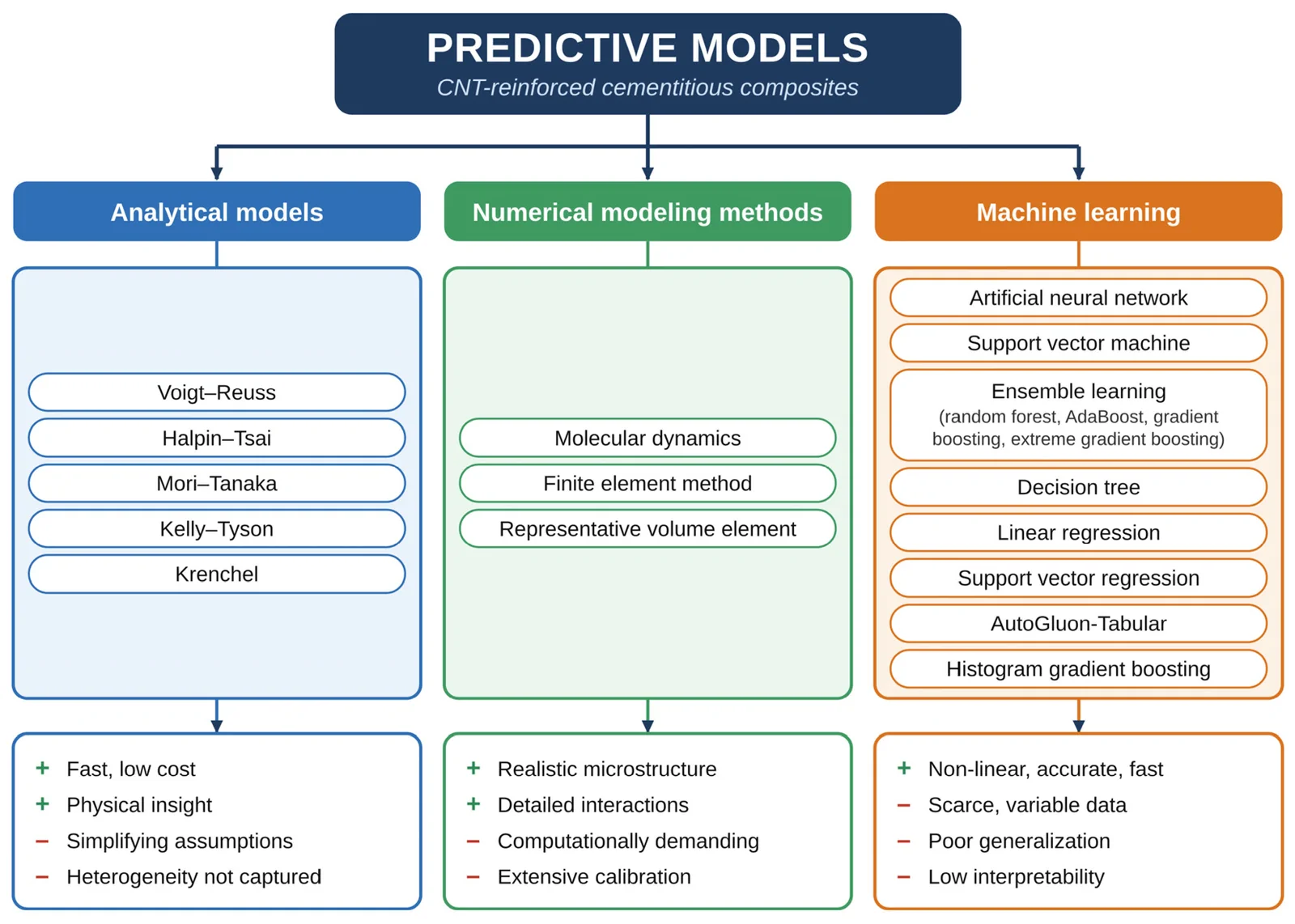
Synthesis of the main predictive models for CNT-reinforced cementitious composites.

**Figure 9:**
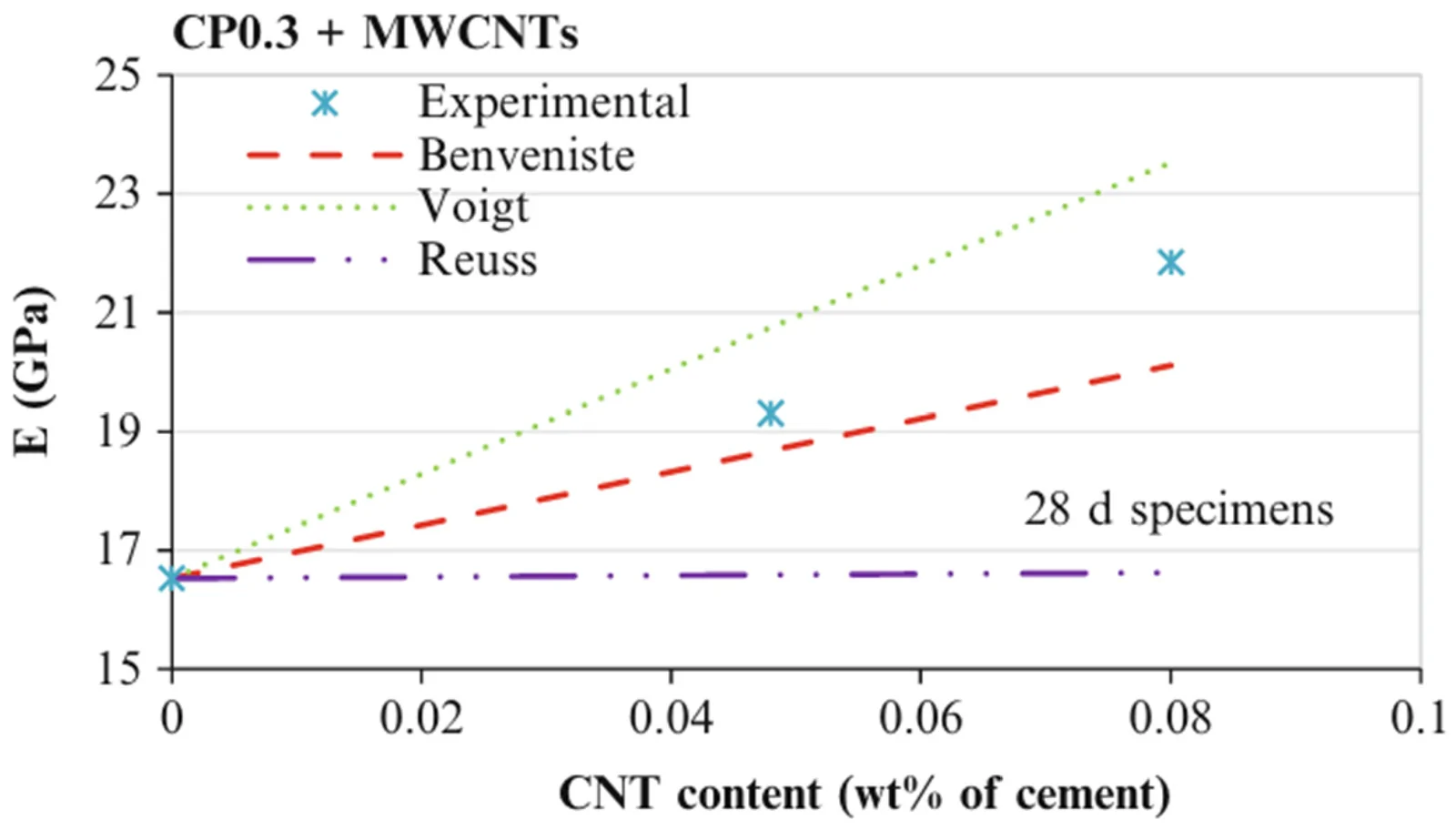
Comparison between predicted and experimental Young’s modulus values of cement pastes reinforced with MWCNTs [85].

**Figure 10:**
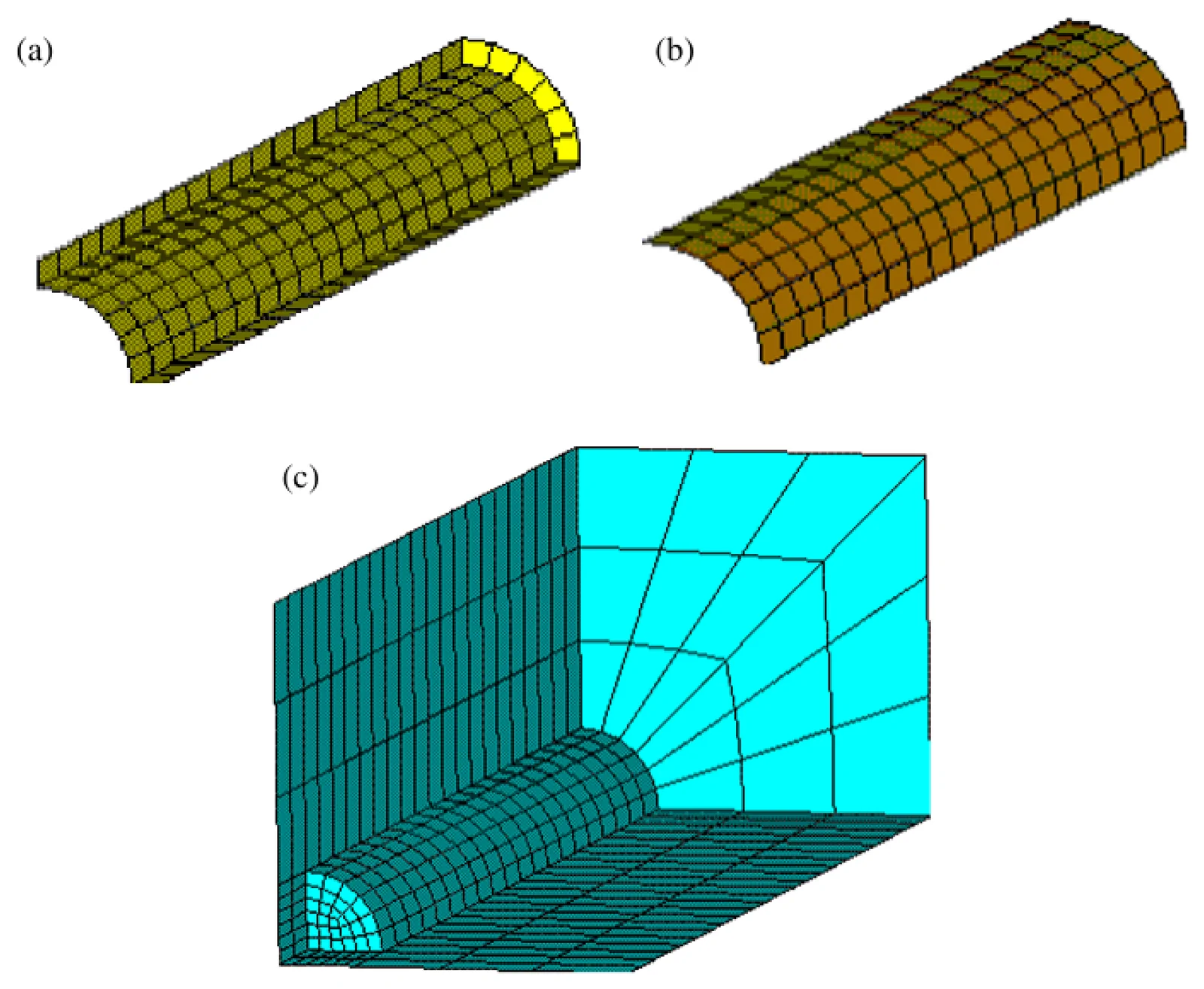
Fundamental components of the composite used for the RVE model: (**a**) embedded CNT, (**b**) CNT–matrix interface and (**c**) cement matrix [92].

**Figure 11:**
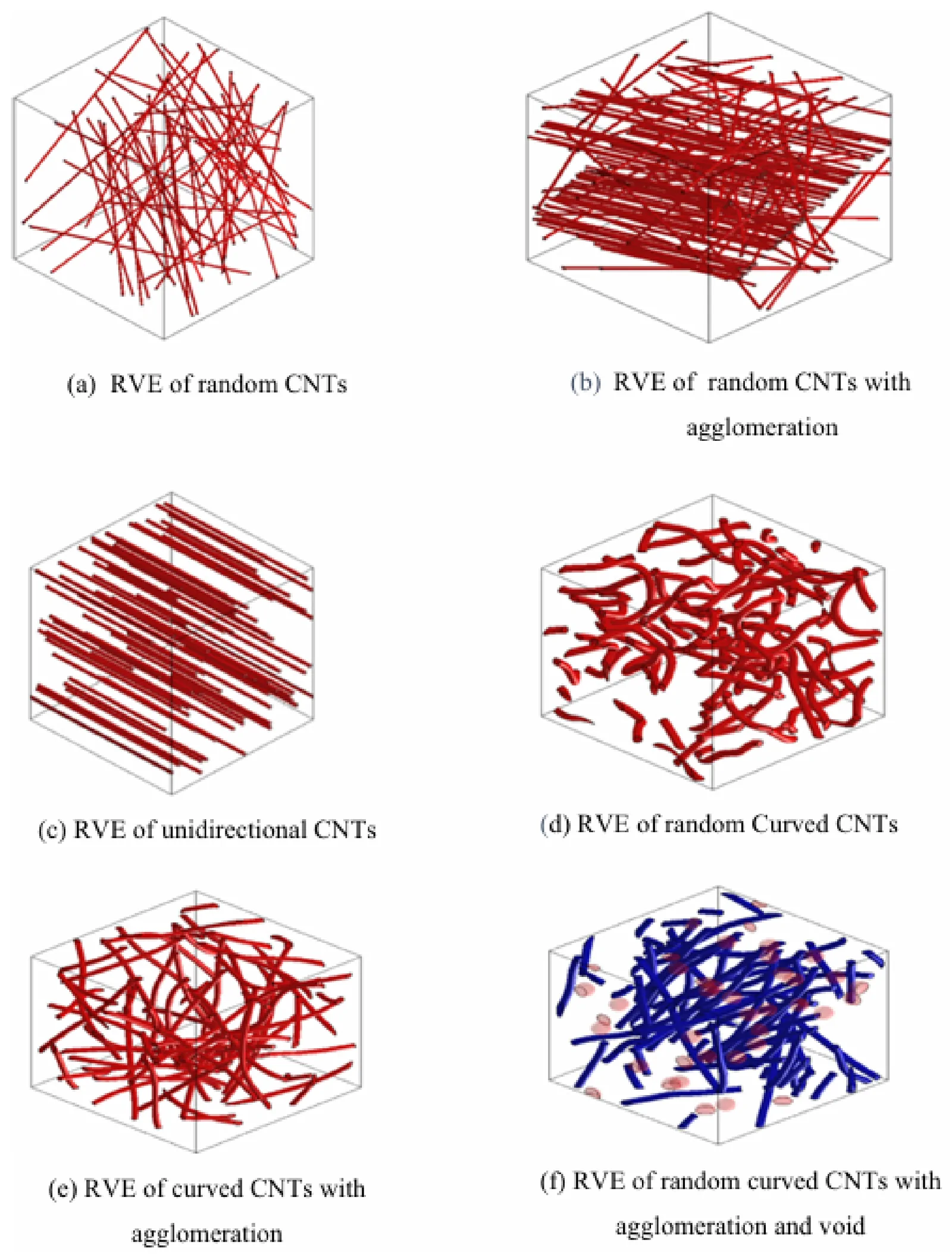
RVE models of (**a**) random CNTs, (**b**) random CNTs with agglomeration, (**c**) unidirectional CNTs, (**d**) random curved CNTs, (**e**) curved CNTs with agglomeration and (**f**) random curved CNTs with agglomeration and voids [91].

**Figure 12:**
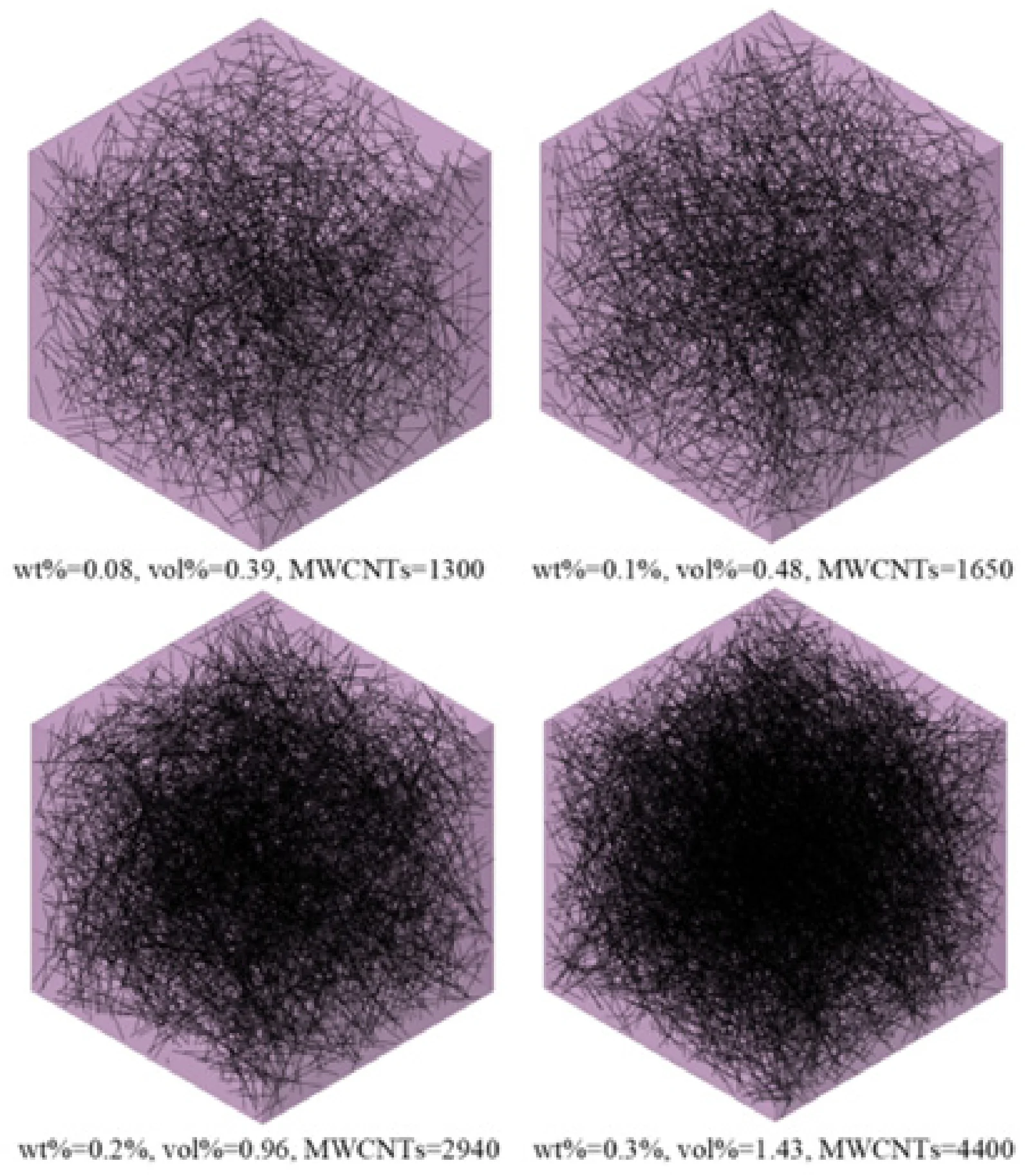
RVE models for numerical simulation of MWCNT-reinforced cement composites [94].

**Figure 13:**
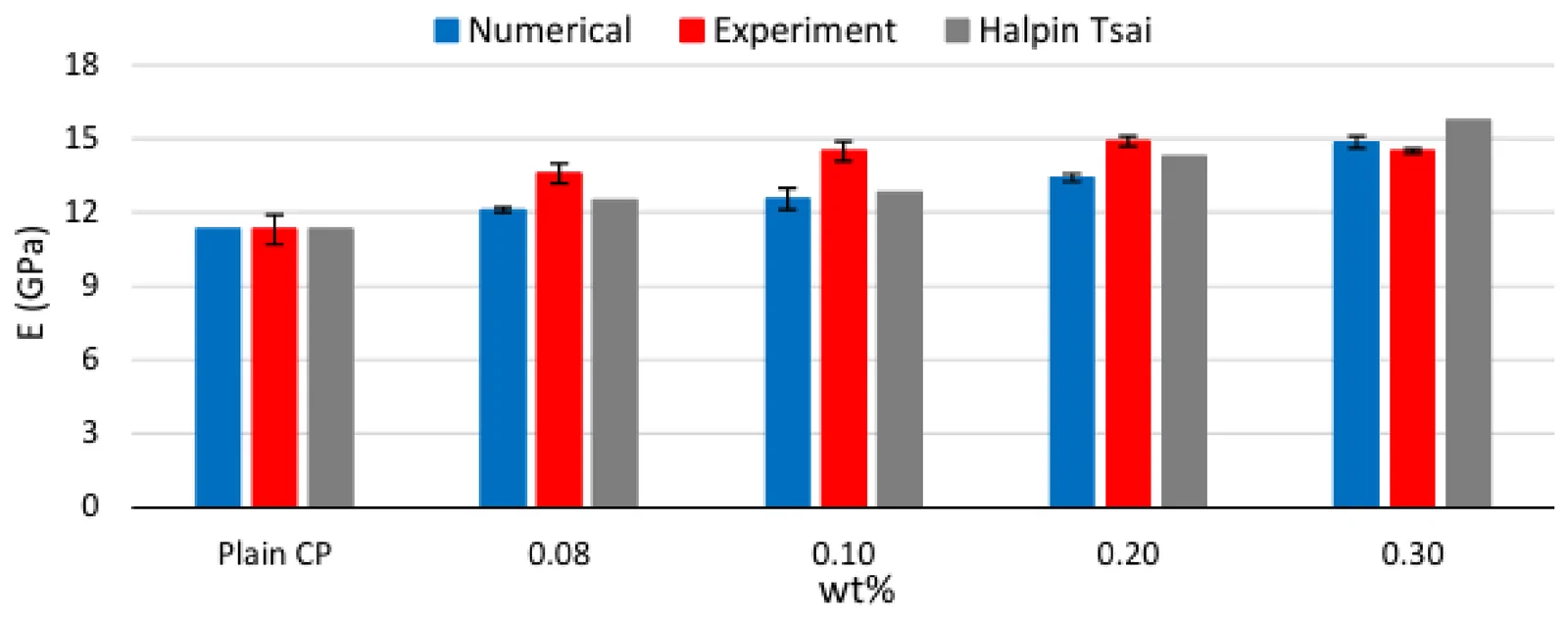
Comparison between numerical, experimental and Halpin–Tsai predicted Young’s modulus values for different MWCNT concentrations [94].

**Figure 14:**
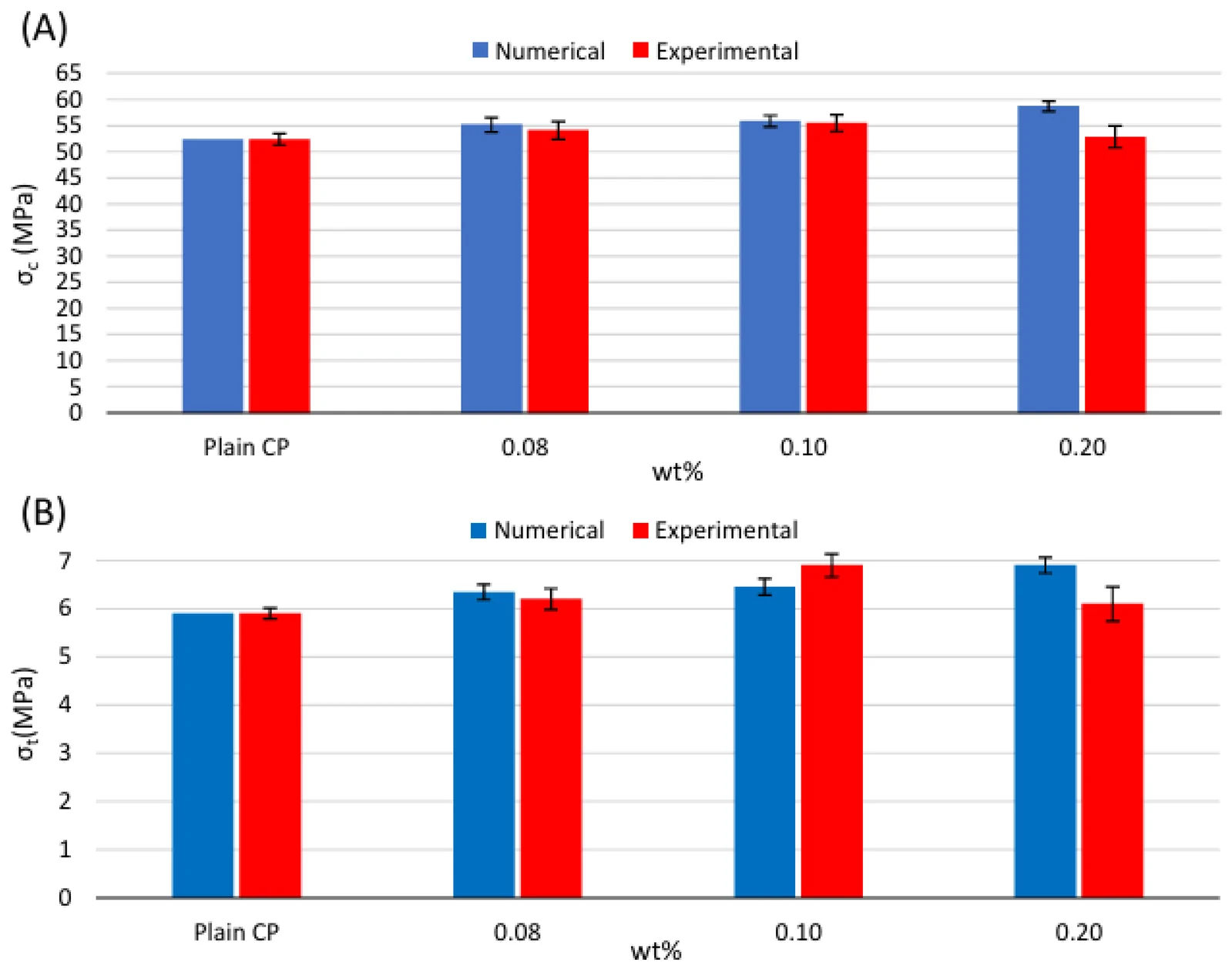
Comparison between numerical and experimental (**A**) compressive and (**B**) tensile strengths for different MWCNT concentrations [94].

**Figure 15:**
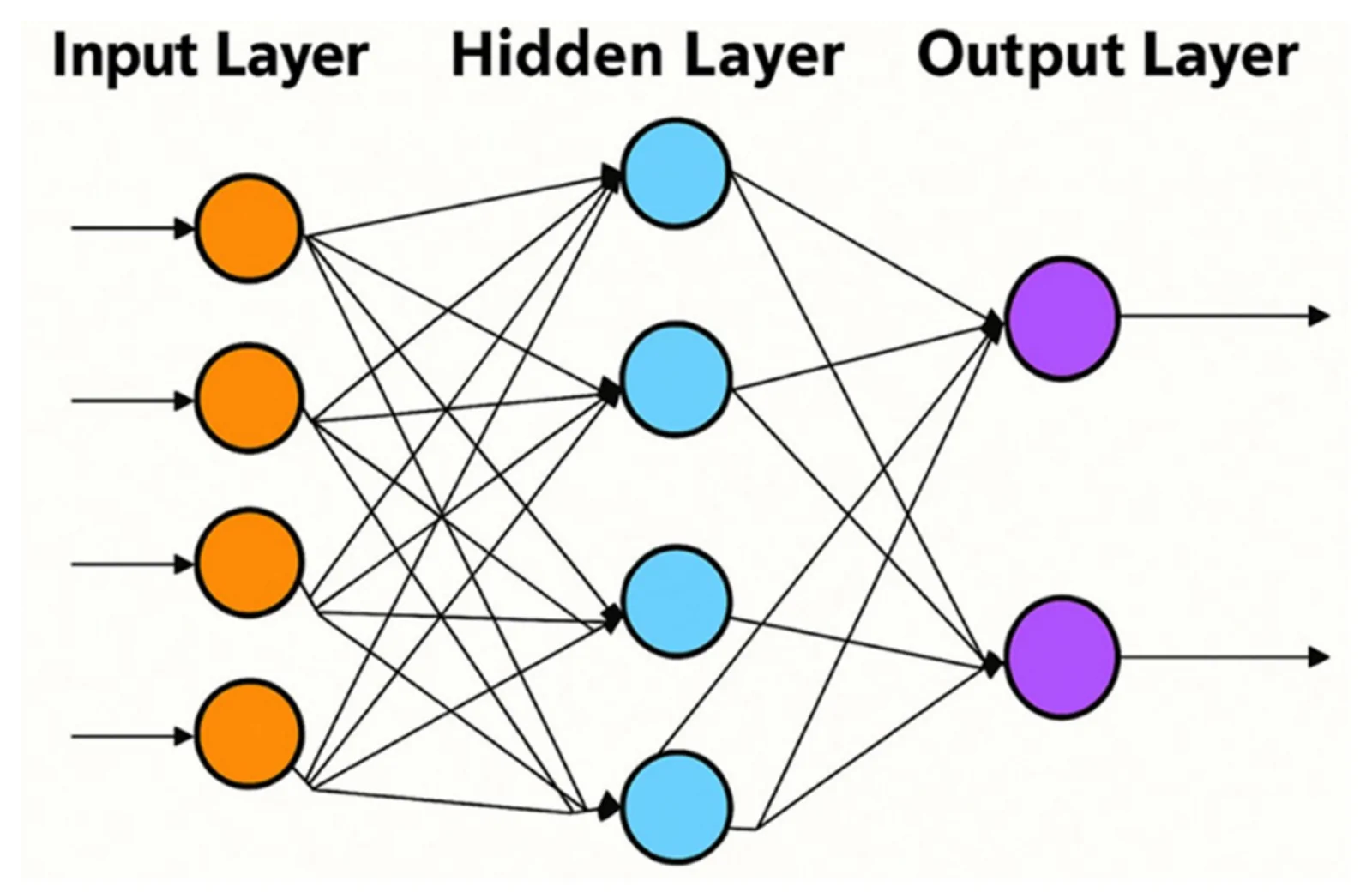
Typical ANN architecture [96].

**Figure 16:**
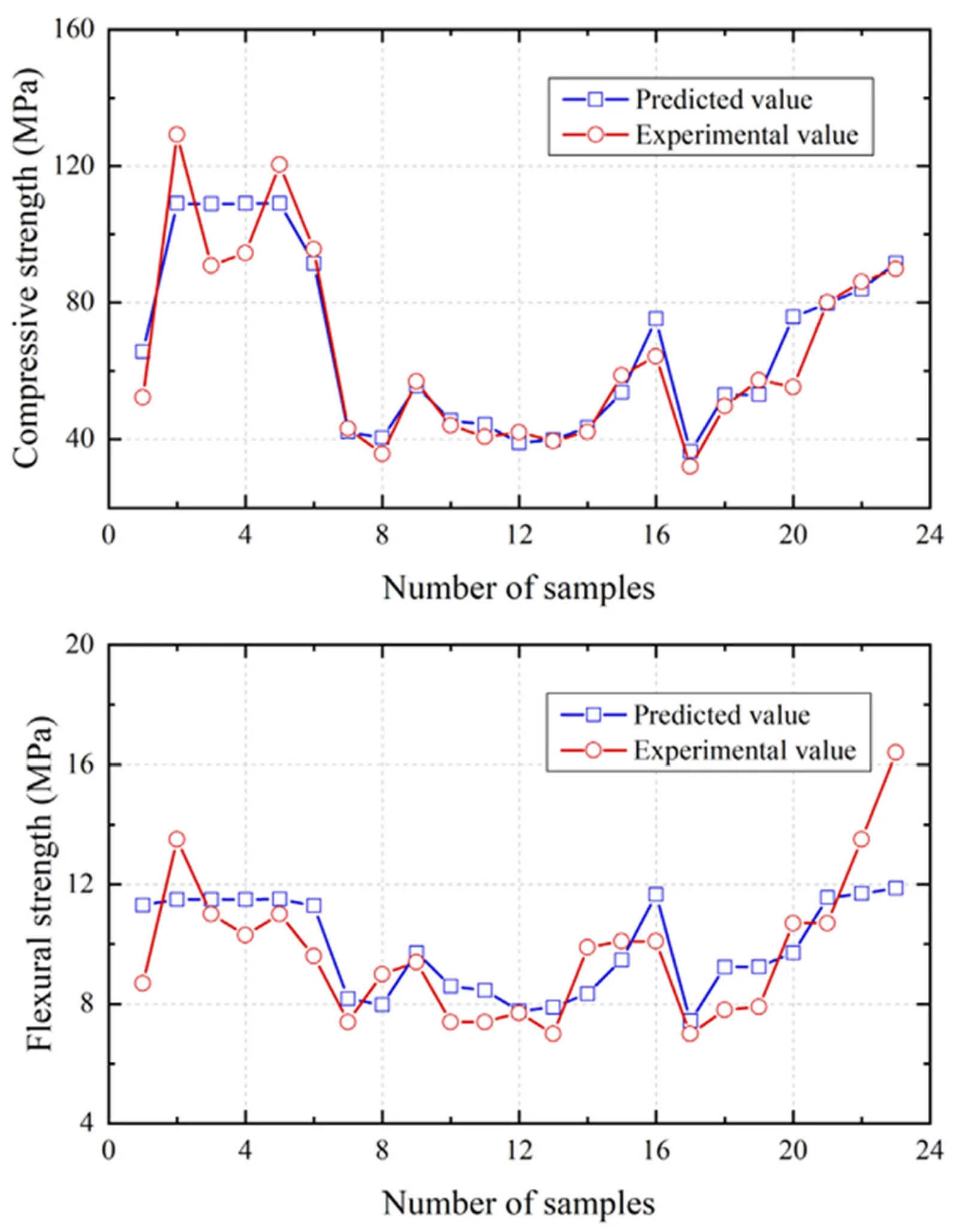
Test results for ANN using the Tanh activation function and an Adadelta optimizer [97].

**Figure 17:**
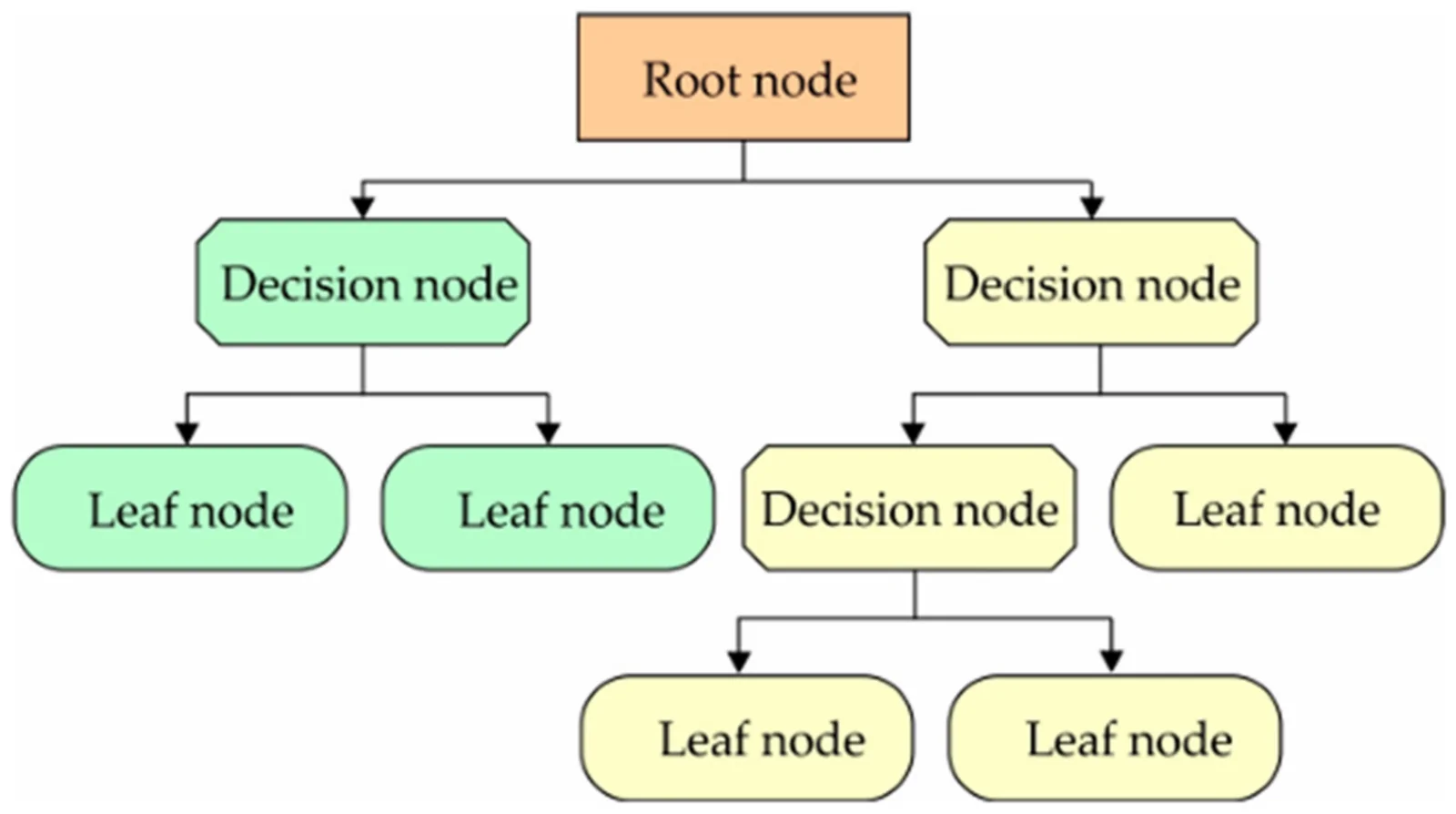
Schematic representation of the DT model [99].

**Figure 18:**
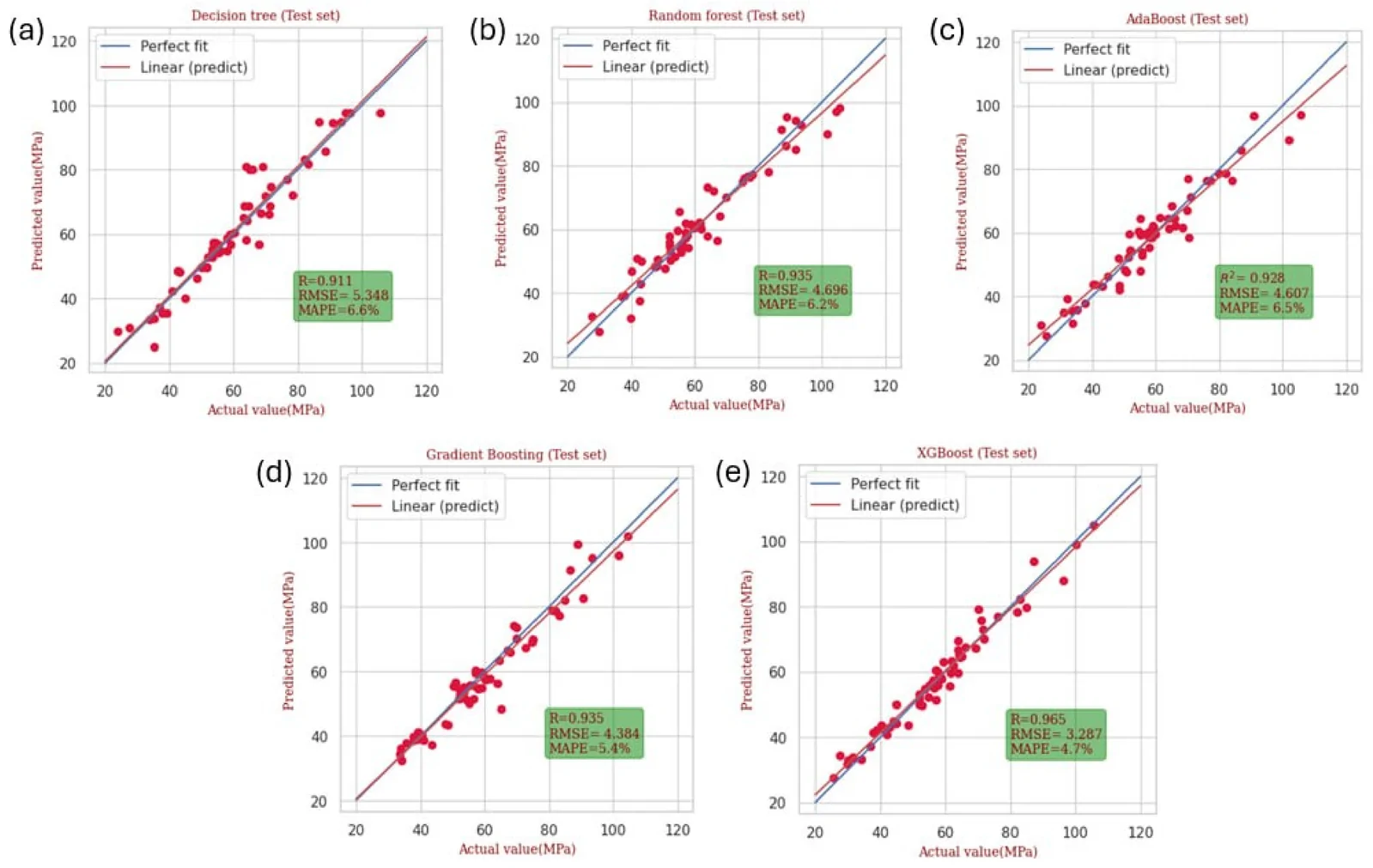
Comparison between predicted and experimental compressive strength employing (**a**) decision tree, (**b**) random forest, (**c**) AdaBoost, (**d**) gradient boosting and (**e**) XGBoost algorithms [98].

**Figure 19:**
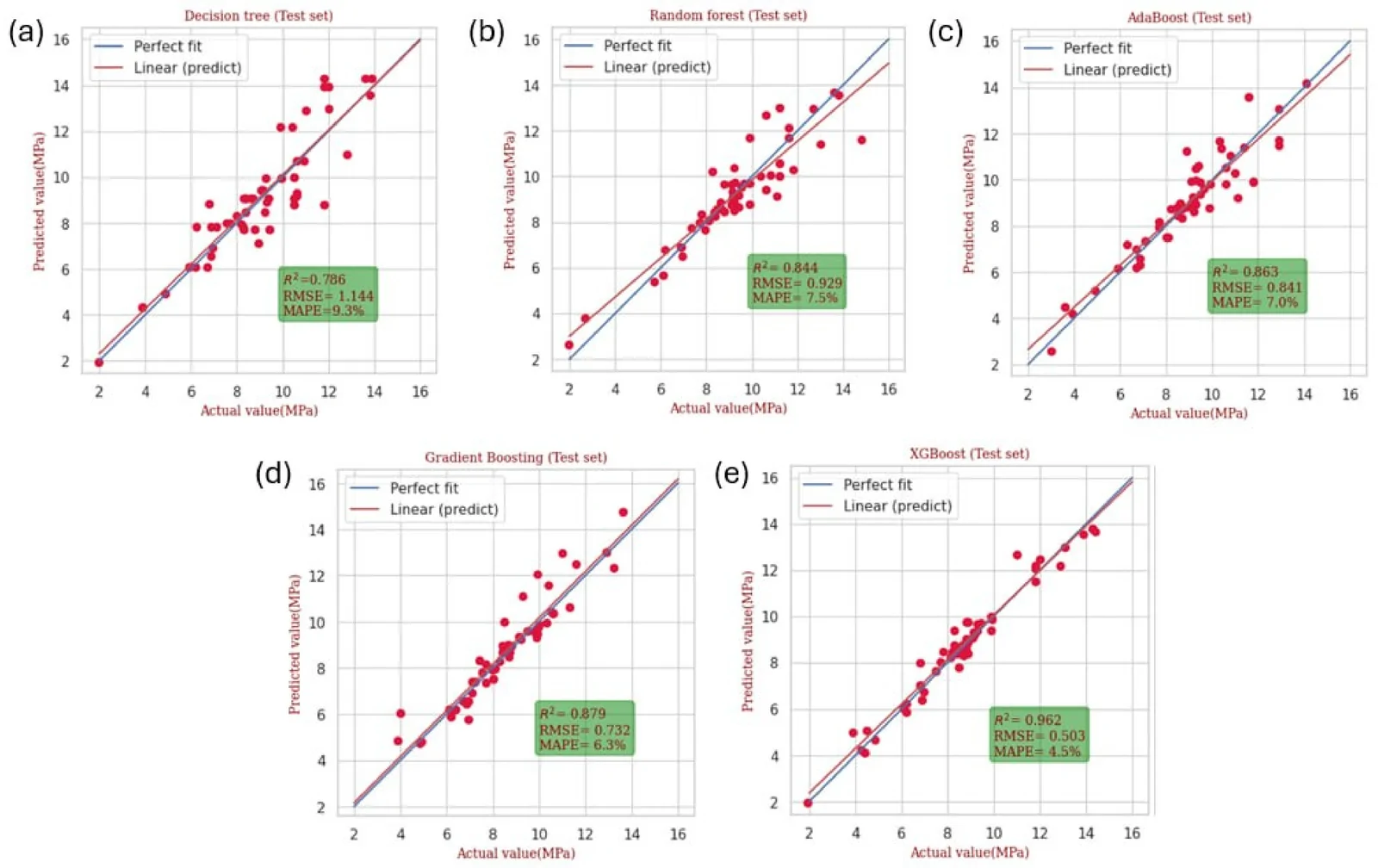
Comparison between predicted and experimental flexural strength employing (**a**) decision tree, (**b**) random forest, (**c**) AdaBoost, (**d**) gradient boosting and (**e**) XGBoost algorithms [98].

**Figure 20:**
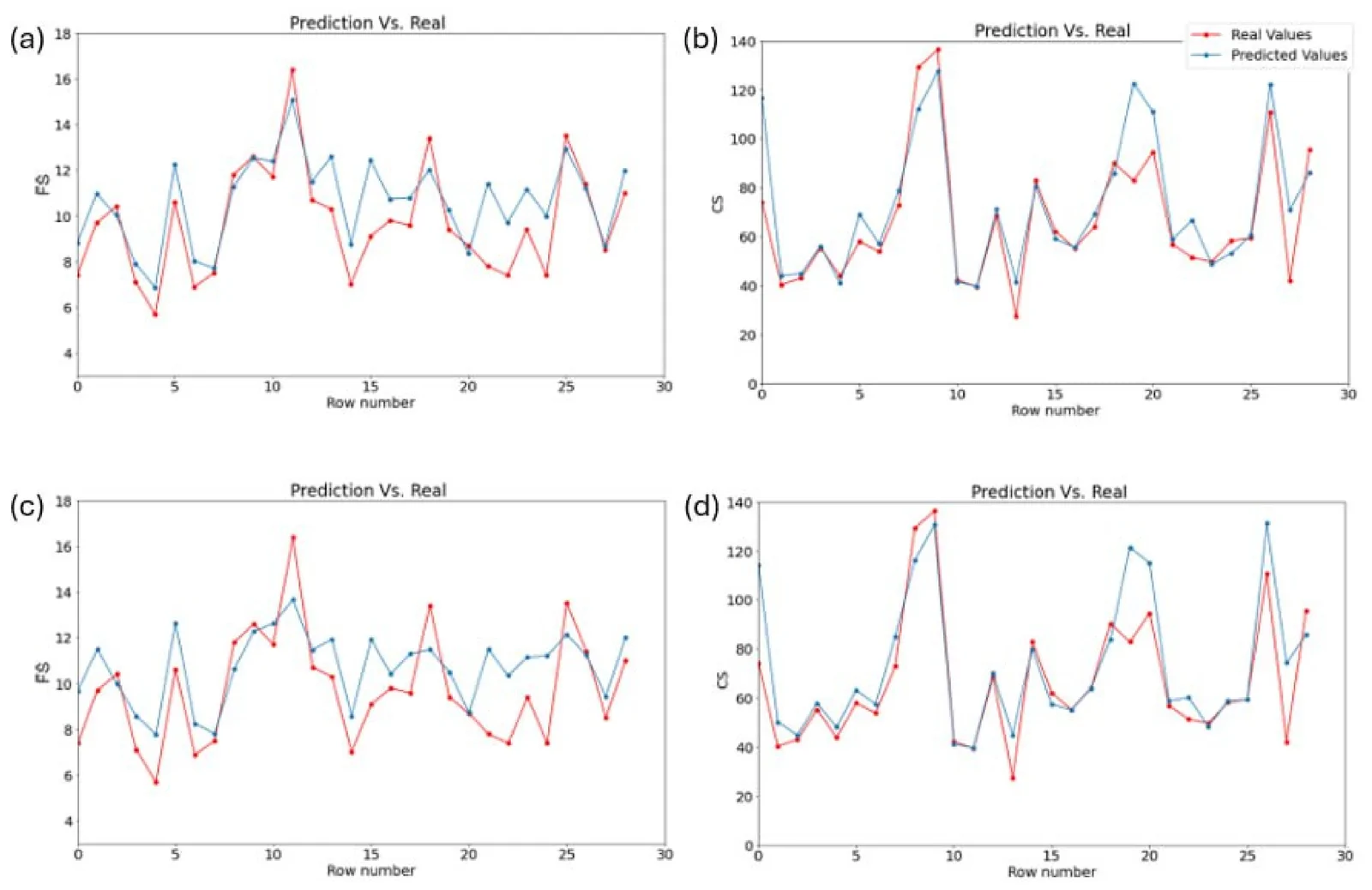
Testing results for each predictive model. (**a**) RF—flexural strength, (**b**) GB—flexural strength, (**c**) RF—compressive strength and (**d**) GB—compressive strength [100].

**Figure 21:**
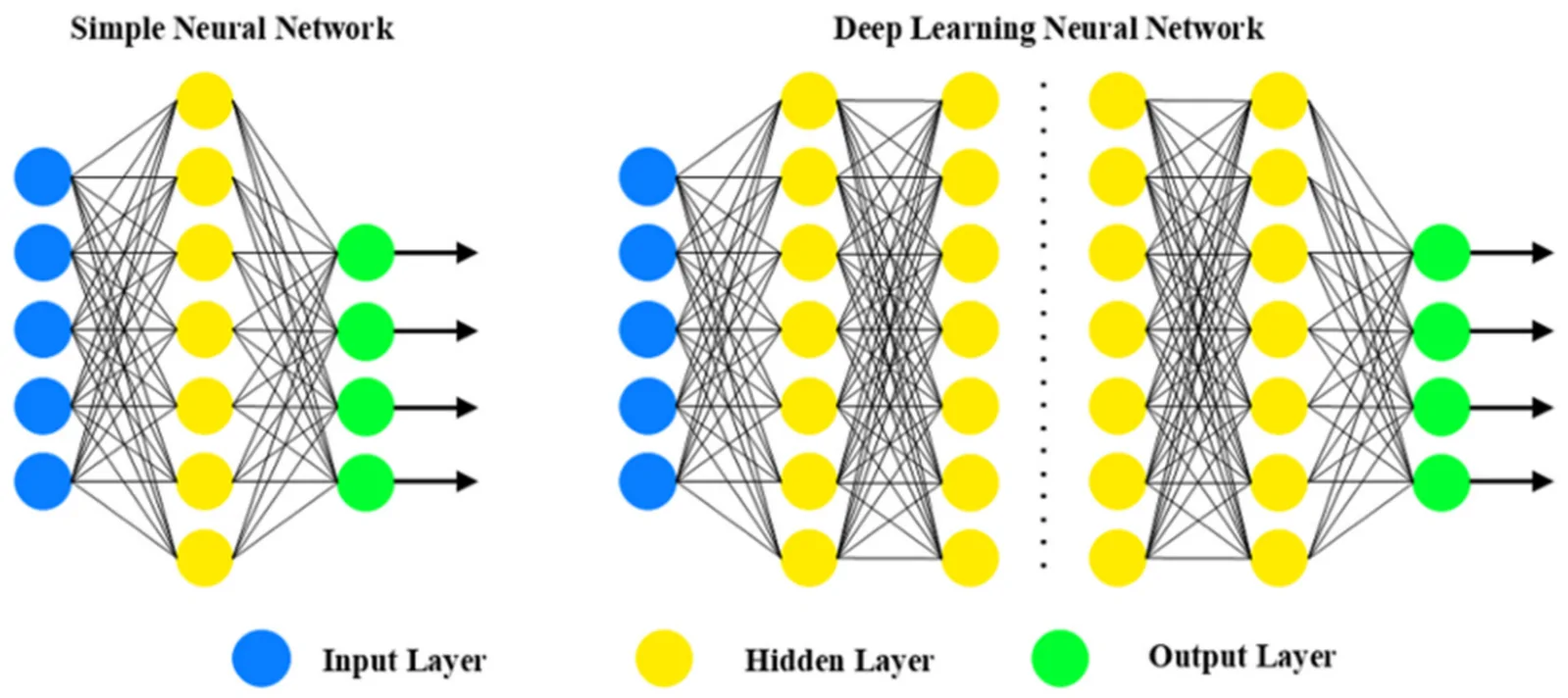
Comparison between simple and deep learning neural networks [105].

**Figure 22:**
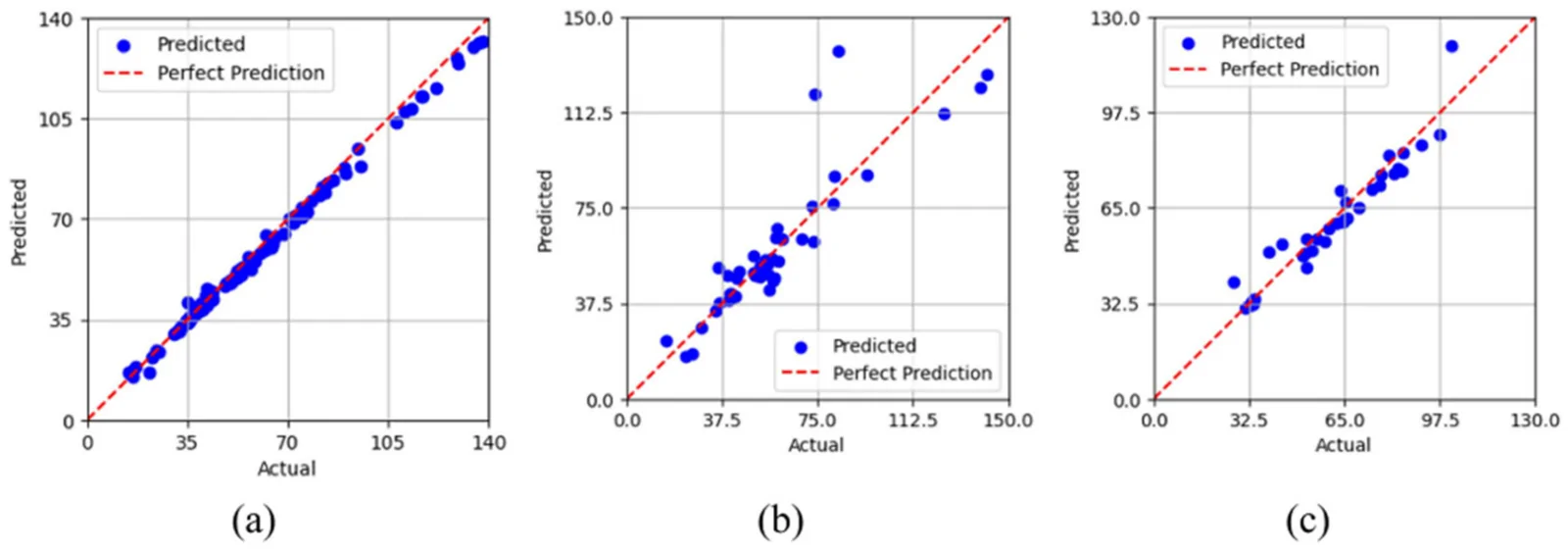
Comparison between predicted and actual compressive strength: (**a**) training, (**b**) testing and (**c**) validation datasets [104].

**Figure 23:**
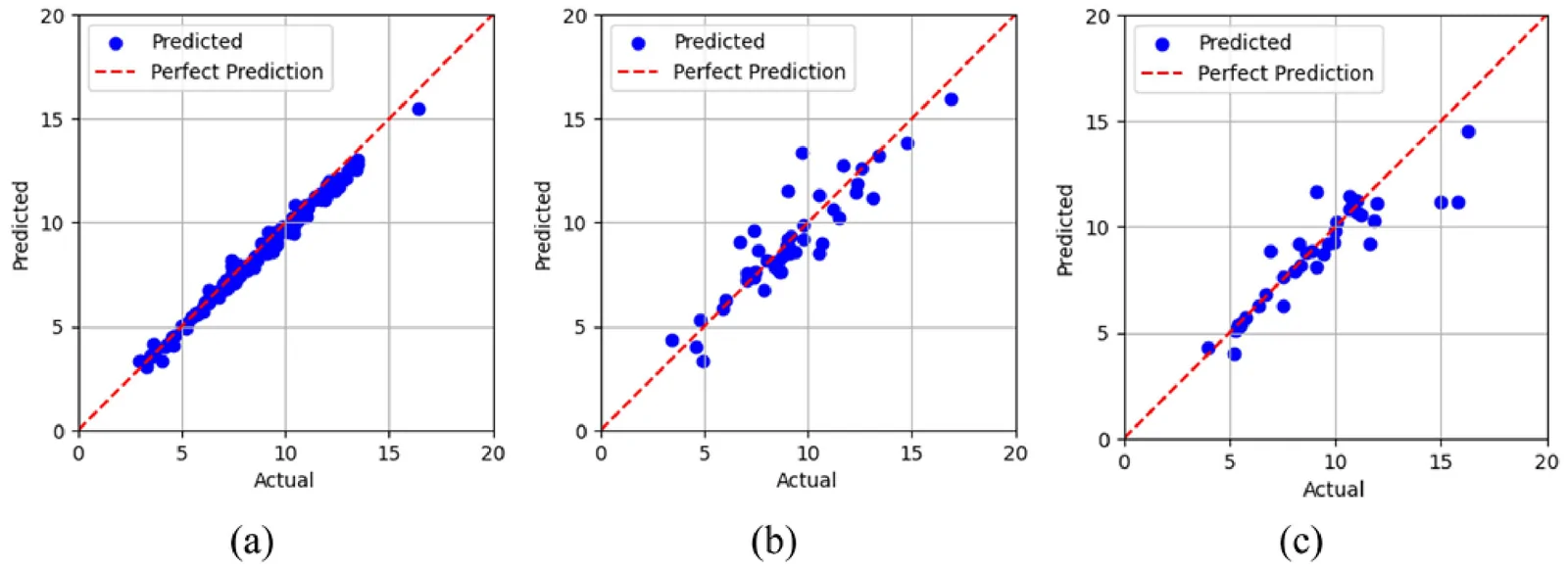
Comparison between predicted and actual flexural strength: (**a**) training, (**b**) testing and (**c**) validation datasets [104].

**Table 1:** Experimental conditions and mechanical performance of CNT-reinforced cementitious composites.

Ref.	Cement Type	CNT Type	Dosage [wt% Cement]	*w*/*c*/*s*	Dispersion Procedure	Mechanical Effect	Proposed Mechanism
**H_2_SO_4_ and HNO_3_ functionalized CNTs**							
[40]	OPC	MWCNTs	2	0.45:1:1.5	Mechanical mixing	CS: +19% FS: +25%	Improved interfacial interaction with hydration products
[58]	OPC	MWCNTs	0.1	0.4:1:0 + silica fume	Surfactant addition	CS: +30% TS: +30%	Carboxyl surface groups interact with hydration products
[62]	OPC	FWCNTs	0.03	0.4:1:0	Ultrasonication	CS: +97%	Carboxyl/hydroxyl groups bond with hydration products
[64]	Class G	MWCNTs	0.1	0.45:1:0	Ultrasonication	Improvement up to 90 min of treatment; decline after 120 min.	Dispersion vs. CNT damage trade-off
[65]	CEM II/A-S R	MWCNTs	0.5	0.4:1:NR	Ultrasonication	Compression resistance: −86%	Hydrophilicity possibly interferes with hydration
**Pristine vs. acid-treated CNTs**							
[55]	OPC	MWCNTs	0.05–0.1	0.45:1:0	Surfactant addition	Acid-treated: lower FS and CS gain than that of pristine	Poor dispersion; increased ettringite
[66]	OPC	MWCNTs	0.04–0.2	0.4:1:0	Ultrasonication	FS higher with pristine	Ettringite formation
**Pristine CNTs**							
[68]	OPC	MWCNTs (CVD-grown)	0.1–1	0.46:1:0	Ultrasonication	FS: +44%; *K*_IC_: +79.7%	Crack-bridging by well-dispersed CNTs

*w*/*c*/*s*: water-to-cement-to-sand ratio; NR: not reported in the reviewed source; CS: compressive strength; FS: flexural strength; TS: tensile strength.

**Table 2:** FEM simulation results.

RVE Configuration	Young’s Modulus of Cement Composites [MPa]	Error [%]
Random CNTs	25,164	9.6
Random CNTs with agglomeration	24,978	8.8
Random curved CNTs	23,865	3.9
Random curved CNTs with agglomeration	23,663	3.0
Random curved CNTs with agglomeration and voids	22,683	−1.2
Aligned random CNTs	36,973	61

**Table 3:** Comparison of the models’ input and output parameters.

	Ref.							
	[97]	[98]	[100]	[101]	[102]	[103]	[104]	[106]
**Input variables**								
**Mix design**								
*w*/*c* ratio	•	•	•	•	•	•		•
*s*/*c* ratio		•		•		•		•
Cement type	•		•		•		•	
**CNT characteristics**								
Geometry ^a^	•	•	•	•	•	•	•	•
Content	•	•	•	•	•	•		•
Surface condition/functionalization method	•	•	•	•	•		•	•
**Dispersion**								
Dispersion method	•	•	•		•		•	
Surfactant type		•		•				
Surfactant/CNT ratio				•		•		•
Ultrasonication ^b^								•
Ultrasonication time				•		•		•
**Curing and testing**								
Curing age	•	•	•	•		•	•	•
Curing temperature	•		•		•		•	
Specimen size				•	•			•
**Control specimen properties**								
Compressive strength		•			•			
Flexural strength		•						•
Elastic modulus								•
**Other ^c^**								•
**Output variables**								
Compressive strength	•	•	•	•	•		•	
Flexural strength	•	•	•			•	•	•
Elastic modulus						•		•

^a^ Length, diameter, aspect ratio. ^b^ Energy, intensity, amplitude. ^c^ Sand type, mixing speed, CNT purity, testing condition.

**Table 4:** Summary of the performance metrics reported for machine-learning models predicting the flexural strength of CNT-reinforced cementitious composites.

Ref.	Material System	ML Model	Performance Metrics				
			R^2^	MAE	MSE	RMSE	MAPE
[97]	Cement paste	ANN	0.846	-	56.123	-	-
		SVM	0.900	-	121.077	-	-
[98]	Cement paste and mortar	DT	0.782	0.844	1.329	1.142	0.102
		RF	0.864	0.671	0.833	0.906	0.086
		AdaBoost	0.865	0.669	0.828	0.903	0.082
		GB	0.911	0.504	0.554	0.732	0.062
		XBG	0.920	0.468	0.480	0.684	0.058
[100]	Cement paste	RF	0.68	1.35	3.09	-	-
		GB	0.69	1.18	2.95	-	-
[103]	Cement paste and mortar	ANN	0.78	1.08	-	1.30	-
		SVR	0.78	1.07	-	1.31	-
		HGB	0.79	1.01	-	1.31	-
[104]	Cement paste and mortar	DL	0.768	7.476	12.707	-	-
[106]	Cement paste and mortar	DNN	0.925	0.517	0.390	0.625	0.082
		Ensemble-bagging	0.899	0.592	0.524	0.724	0.090
		SVR	0.802	0.841	1.024	1.012	0.156

## Data Availability

No new data were created or analyzed in this study. Data sharing is not applicable to this article.
